# Sustainable strategies for green supply chain within the platform economy consider subsidies and marketing efforts

**DOI:** 10.1371/journal.pone.0292349

**Published:** 2023-11-28

**Authors:** Weisi Zhang, Yiting Wu, Rui Luo, Yu Wang

**Affiliations:** 1 Logistics Research Center, Shanghai Maritime University, Shanghai, China; 2 School of Management, Fudan University, Shanghai, China; Central Queensland University, AUSTRALIA

## Abstract

As consumers’ green awareness continues to grow, the level of a product’s eco-friendliness and the quality of its marketing have become significant factors in shaping consumers’ purchasing decisions. The power structures within the supply chain, as well as corresponding government subsidy policies, are also key elements influencing sustainable strategies for the green supply chain. In a green supply chain comprising one manufacturer and one e-commerce platform, two sales models exist within the online e-commerce platforms: reselling and agency selling. This paper establishes and analyzes three distinct Stackelberg game models, namely: manufacturer-led model without subsidy (*bm*), manufacturer-led model with subsidy (*sm*), and platform-led model with subsidy (*sp*). The results are shown as follows, with the rise of consumers’ environmental awareness, more consumers opt for green products, inspiring the manufacturer to increase its optimal greenness and platform to enhance its optimal marketing efforts level. Notably, government subsidies provide a significant stimulus. An increase in the green technology cost coefficient leads to a decline in the manufacturer’s profits across all three modes. Intriguingly, the manufacturer’s profits are always highest in the sp mode. As the marketing efforts cost coefficient increases, the platform’s profits decrease in the bm and sm modes. In contrast, in the sp mode, the platform’s profits increase rather than a decrease. The choice of mode primarily depends on the platform’s marketing efforts cost coefficient. When this coefficient exceeds a threshold, the platform chooses the sp mode. However, due to the relatively low marketing efficiency in this scenario, the manufacturer prefers the sm mode. For the government, the sp mode involves agency selling, serves as an effective mechanism to redistribute subsidies, thereby yielding the maximum social welfare benefits. Management insights are provided for the manufacturer and platform managers to make decisions about the degree of greenness and marketing efforts level, along with insights for governments to optimize subsidy policies.

## 1. Introduction

In recent years, consumers’ attitudes towards sustainability have become increasingly "green". The Global Sustainability Study (2021) indicates that 85% of consumers exhibit this trend, although there are variations across different countries and industries. Therefore, manufacturers are encouraged to consider the potential impacts that their products, services, and supply chains may have on human health and the environment. The emphasis on green products is intrinsically linked to carbon emission reduction. As products are designed and manufactured to be more environmentally friendly, their carbon footprint decreases. This direct correlation between the greenness of a product and its carbon emissions is pivotal in the global effort to combat climate change. With the strong growth of online e-commerce platforms, two popular sales formats have emerged: reselling and agency selling [1]. In the reselling mode, e-commerce platforms act as sellers, purchasing products from suppliers and reselling them to consumers [2], such as Walmart.com, Vipshop.com, Gome.com, and Suning.com. Another increasingly popular online business format is agency selling. Under this format, retail platforms do not purchase products from suppliers and then resell them to consumers, but instead provide suppliers with access to consumer traffic on the platform and charge the commission fee. In the agency selling format, suppliers can sell their products directly to consumers, and the platform will charge the commission fee for each unit of sales [3]. For example, one of the world’s largest retail platforms, Alibaba, has provided a marketplace for independent suppliers to sell a wide range of products directly to consumers. The Chinese Ministry of Commerce introduced regulations in 2021 (Office of the Ministry of Commerce). Support e-commerce platforms to expand sales of green, energy-saving, environmental protection and other products, set up special zones for the sale of green products, strengthen centralized display and advertising, and guide consumers to buy green products. The promotion of green products within sustainable supply chains is a testament to the industry’s commitment to carbon emission reduction. By ensuring that products are designed, manufactured, and marketed with sustainability in mind, the entire supply chain contributes to the global carbon emission reduction efforts. Companies like Apple Inc. and IKEA are leading the way in carbon emission reduction initiatives by focusing on the production of greener products. Such products inherently have a reduced carbon footprint, emphasizing the direct relationship between product greenness and carbon emission reduction. For example, Apple Inc. has released its "2020 Environmental Progress Report" committing to reducing its carbon emissions by 75% by 2030, with the remaining 25% to be offset through investment in natural environmental protection projects. Besides Apple, other companies are also accelerating their emission reduction plans. According to The Wall Street Journal, multinational corporations such as Microsoft, Nike, Starbucks, Unilever, and Danone are forming a new alliance dedicated to sharing resources and strategies for reducing carbon emissions. The European Union’s "Green Deal" aims to reduce its carbon emissions by 55% by 2030, while the UK government plans to reduce its carbon emissions by 100% by 2050. One of the world’s largest home furnishing retailers, IKEA, is currently reducing its carbon emissions by adopting more environmentally-friendly materials and production methods. Additionally, IKEA is providing sustainability guidance and support to its supply chain partners to promote eco-friendly production and consumption practices. Based on the above analysis, many governments, companies and supply chains are paying more attention to green products to achieve carbon reduction [4, 5]. Green supply chain emission reduction and government subsidy regulation have become urgent and fiery topics, and attracted extensive discussions from the academic and business communities.

This research focuses on the sustainability of green supply chain strategies in the platform economy, which is motivated by the urgent need to address global warming caused by carbon emissions [6]. Within the platform’s supply chain, the environmental performance assessment of manufacturers’ products includes their greenness. In line with this, JD has screened 3,273 suppliers for ecological criteria, and in 2020, 85% of these suppliers were new to the company. The promotion of green consumption has become a hot topic in recent years, with Tmall and 14 brands launching the "Green Merchant Alliance" in 2021 to promote sustainable practices (All-China Federation of Environmental Protection). The increasing awareness and adoption of sustainable consumption have led to a rise in the public’s green lifestyle. According to a report by New York University, sustainable products accounted for 17.0% of the market share in 2021, a 3.3 percentage point increase from 2015. Additionally, sustainable products have grown 2.7 times faster than products that are not marketed as sustainable and have continued to grow even during the COVID-19 pandemic (Sustainable Market Share Index^™^). With consumers’ growing demand for sustainability, platforms can play a crucial role in providing and promoting environmentally friendly products and services. They can facilitate this by displaying the environmental footprint of goods and services, motivating consumers to choose green alternatives, and educating the public about sustainable consumption. However, platforms must be cautious in their sustainability strategies to avoid resource waste and profit decline. While green products offer a path towards sustainable development, their production and distribution can also pose new challenges that need to be addressed.

There has been significant research conducted in the field of green supply chain management. Governments have introduced various subsidy programs to encourage green development, including green non-subsidy (GNS), green product subsidy (GPS), and green innovation subsidy (GIS) [7]. These programs aim to incentivize manufacturers to adopt sustainable practices and develop green products. With the increasing awareness of environmental issues, there has been a growing demand for green products among consumers. Green products offer additional utility to environmentally conscious consumers compared to brown products, creating significant opportunities for green product development [8]. Such products include organic food, eco-friendly batteries, energy-efficient appliances, eco-friendly paints, and new energy vehicles, among others. To manufacture green products, conventional manufacturers must invest in new machines, green design, and R&D for production. It is worth noting that the production cost of green products is generally higher compared to brown products. To promote green products, platforms need to invest in upgrading their green marketing efforts, as consumers’ actual green product purchase behavior is influenced by the platforms’ efforts [9]. For example, Walmart takes a series of green marketing efforts for the green and energy-efficient products produced by P&G. While previous studies have discussed government green subsidies and green marketing efforts [10–13], there is still a lack of clarity regarding green supply chain sustainable strategies within the platform economy. One of the unique characteristics of the platform economy is that platforms and manufacturers have different market power structures. Platforms can reselling or agency selling green products, which presents opportunities and challenges for green supply chain sustainability strategies. Thus, our research aims to address this gap and provide original theoretical contributions to the field.

In the current e-commerce industry, there are two general sales models that platforms use. The first model is the reselling model, in which the platform purchases products from manufacturers, determines the selling price, and sells the products directly to consumers through its own platform, such as the platform’s own store. The second model is the agency selling mode, in which the manufacturer sells products through the e-commerce platform and determines the product price, while the platform plays an intermediary role and charges a certain fee. This model is commonly used by various official flagship stores on the platform. When shopping online at websites like JD Mall, Taobao, Amazon, and Suning Tesco, consumers have the option to purchase from either the manufacturer’s official flagship store or the platform’s own store. To promote green development, the government has introduced a green innovation subsidy (GIS) program for dominant companies, whereby the manufacturer produces green products, and the platform provides green marketing efforts. However, the government’s aim of maximizing social welfare may not align with firms whose primary goal is profit maximization. Therefore, firms may prefer different sustainable strategies based on their cost-benefit trade-offs. This highlights the importance of understanding the trade-offs between green supply chain sustainable strategies and the financial goals of both platforms and manufacturers within the platform economy.

Based on the challenges related to the production and distribution of green products discussed above, the objective of this research is to investigate the following critical managerial questions:

1. How can the government determine the optimal proportion of green subsidies under different power structures?
2. How does the variation of green cost coefficients affect the profit of both the manufacturer and the platform?
3. How can the optimal model be selected for different scenarios, and is it possible to achieve Pareto improvement?

To answer these questions, this paper constructs a supply chain consisting of one manufacturer and one platform (e-tailer). The e-commerce platform engage in reselling or agency selling. We take the non-subsidized manufacturer-led scenario as the benchmark model (*bm*). Under government subsidies, we consider the manufacturer-led model (*sm*) and the platform-led model (*sp*), respectively. Previous studies have shown that as the cost coefficient of green technology increases, the profits of both the manufacturer and the platform decline. In contrast, we find that the platform’s profit rises in the *sp* model as the cost coefficient increases. The manufacturer and platform choose the *sp* and *sm* model respectively, when the cost coefficient for marketing efforts is negligible. Moreover, our findings show that *sm* model is the best selection when the cost coefficient is moderate. Furthermore, although our study is motivated by sustainable strategies for a green supply chain within the platform economy, our main findings may have important theoretical and managerial implications for other scenarios, such as channel selection and market competition.

The main contributions of this paper are manifested in the following aspects: First, examining the sustainable strategies of green supply chains. Considering the increasing green consciousness among consumers, this paper proposes a sustainable strategy that combines product greenness with marketing efforts. This is the first application of such a strategy in the context of platform economy, providing new ideas for realizing green supply chains. Second, different from existing research, this paper focuses on Green Innovation Subsidies (GIS), which target dominant enterprises in the supply chain, expanding the assumptions of government green subsidy regulation. Unlike existing studies that only consider the form of government subsidies [6, 10, 14], this paper provides a new perspective, offering references for the government to formulate green subsidy policies. Third, investigating the impact of reselling and agency selling on different market power structures under the platform economy. Unlike previous studies that only focus on traditional market power structures, this paper considers the changing market environment resulting from the rapid development of e-commerce, better reflecting the reality of the market. Overall, this paper proposes a sustainable green supply chain strategy, explores the impact of government green subsidy policies and market power structures on the supply chain, and provides new ideas for researching green supply chains.

The rest of the paper is organized as follows. Section 2 presents a comprehensive review of the relevant literature and contextualizes the study. Section 3 introduces the model framework, notation, and assumptions used in this paper. Section 4 presents the non-subsidized manufacturer-led benchmark model (*bm*), the subsidized manufacturer-led model (*sm*), and the subsidized platform-led model (*sp*), along with the equilibrium results. Section 5 analyzes and compares the three models, investigates the effects of key factors using numerical experiments. Section 6 summarizes the main findings and highlights the managerial implications and future research directions. The S1 Appendix provides all proofs.

## 2. Literature review

The research is focused on three main areas of literature: the green supply chain (including consumers’ green awareness, production of green products, and marketing efforts for green products), government policies related to green subsidies (including non-subsidized green initiatives, green product subsidies, and green innovation subsidies), and the e-commerce platform economy (including market power structures, reselling, and agency selling). A review of the relevant literature has been conducted to identify the current research gap in these areas.

With the increasing requirements for socially sustainable development, consumers’ awareness of environmental protection is constantly increasing, and green products and logistics are attracting attention from all walks of society. Khan et al. [15] employed the Generalized panel Method of Moments and found that green practices can mitigate the harmful effects of logistics operations on environmental sustainability and stimulate economic activity, therefore, many scholars bring consumers’ green awareness and the production of green products into the green supply chain [16–18]. From the existing research, the current research on consumers’ green awareness and green product production in the green supply chain is mainly focused on pricing strategy and its impact on strategy, Rahmani et al. [19] proposed a Stackelberg game approach in the decentralized scenario to studied the pricing problem of the green supply chain under the interruption of market demand, Li et al. [20] considered a dynamic duopoly game to studied the impact of consumers’ green awareness and emission tax on corporate green innovation investment and pricing strategies. In addition, whether consumers purchase green products is directly related to their understanding of such products, which is to some extent influenced by the marketing efforts of the sales platform. Manufacturers are responsible for designing and producing green products, while retailers must exert significant efforts to effectively market green products. Therefore, many scholars also consider the marketing efforts of green products in the research of the green supply chain [21–24], among them, Guo et al. [21] formulated a Stackelberg game model to study the green supply chain of green product design and marketing efforts with sales platform eco-labels, and said that no matter how effective the marketing efforts are, the improvement of the green level of products can benefit enterprises. Kinds of literature construct the Stackelberg differential game model [25, 26] and dual-channel supply chain [22, 27] to explore the impact of factors such as the adjustment of marketing efforts on supply chain stability and profits.

Developing green products requires more research and development investment than conventional products. Thus, obtaining technical funds is a major challenge for enterprises implementing green supply chains. To encourage green product production, it is necessary to explore the need for government intervention in green enterprises through subsidy policies. However, limited government financial resources require appropriate subsidy policy design and impact analysis on enterprises, consumers, and society. Researchers have studied various subsidy programs and their effects. Government subsidies provide incentives for the green transformation of logistics in some industries, such as the pharmaceutical industry [28]. Zhang et al. [29] based on the numerical simulation and coefficient sensitivity analysis, conclusion that the improvement of public awareness of environmental protection, government subsidies also increase, Meng et al. [30] use the research method of Stackelberg game theory to conclusion the government subsidies can reduce the price of green products to a certain extent and effectively promote the sale of green products, In addition, Sajid et al. [31] through the principal axis factor extraction and regression analysis suggests that the government should provide monetary incentives to enterprises to reduce green logistics costs. Gui et al. [32] through evolutionary game theory, government subsidies can encourage supply chain enterprises to reduce carbon emissions. However, subsidies do not always bring benefits, Feng et al. [11] said that subsidies in the remanufacturing mode are ineffective when remanufacturing costs are low, and higher subsidy rates do not always benefit the environment, social welfare and social surplus [33] at the same time. Chen et al. [10] present a three-player game that studied unit production subsidies and innovation efforts subsidies and said that the government should not use these two subsidies for product research and development efforts to reduce costs, Wu et al. [6] studied the carbon trading mechanism under subsidies and the interaction strategy between the government and supply chain enterprises, and finds that in the case of no cost-sharing, the type of decision-making between enterprises has no impact on the level of low-carbon technological innovation and subsidies. [32, 34, 35] through establishing the Stackelberg game analysis framework, design of different scenarios, study of different government subsidy strategies and the impact of government subsidies on the green supply chain.

With the rapid development of e-commerce, more and more consumers choose to buy green products through the e-commerce platform. Some e-commerce platforms promote product promotion and sales through online celebrity endorsements [36], Some e-commerce platforms allow manufacturers to dock directly with their customers but at the same time charge manufacturers a specific commission fee, that is, some scholars are now studying the agency selling of the supply chain [21], agency contracts bring higher profits, while wholesale price contracts bring more remarkable environmental improvement. Abhishek et al. [1] use a stylized theoretical model to research shows that agency selling is more effective than reselling and leads to lower retail prices, but at the same time, electronic retailers eventually hand over control of retail prices to manufacturers. After that, for this model, scholars have carried out a series of studies to explore the optimal operation strategy in the green platform supply chain under different market power structures, considering reselling and agency selling, for example, Wang et al. [22] constructed and analyzed a centralized model and a decentralized model with or without fairness concerns, and proposed a "cost-sharing joint commission" contract to achieve system coordination, Wang et al. [26] combines different sales channels with financing models. As the commission rate increases to a certain level, we can find the threshold of the cost coefficient of emission reduction. When consumers show higher green awareness, manufacturers face lower commission rate and higher profit margins [21]. Zhang et al. [37] constructing two Stackelberg game models suggest that weaker platforms can gain more utility by voluntarily reducing their altruistic preferences when commission rate is low. In addition, Zhang et al. [38] show the dominance of any retailer over the manufacturer under the contract.

In summary, sustainable strategies for green supply chains have garnered significant attention from both practitioners and scholars. The current work is aligned with existing literature, while differing in the following aspects. First, with the growing environmental problems, green sustainability issues are widely concerned, consumers are green conscious [8, 39], there are several literatures that consider the production of green products in order to meet consumers’ green preferences [5, 13, 40]. Consumers’ actual green product purchase behaviour is influenced to some extent by the green marketing efforts of the platform [9, 41]. To the best of our knowledge, this is the first attempt to apply a combination of product greenness and marketing efforts to obtain a sustainable strategy in a platform economy. Second, several studies have focused on the area of government subsidies, since green sustainable strategies require significant investments. Governments mainly consider green non-subsidy (GNS), green product subsidy (GPS) and green innovation subsidy (GIS) these three government subsidy programs [7]. For the allocation of government subsidies, there are also several studies to develop contractual mechanisms to achieve coordination of the green supply chain [13, 24, 25]. We focus on green innovation subsidies (GIS) and discuss government subsidies to the dominant firm in the supply chain, extending the assumption of governments’ green subsidy regulation. Finally, the rapid development of e-commerce and platform economy has made reselling and agency selling to be the main sales channels [12, 42]. Unlike previous studies that focused on the encroachment strategies of suppliers or manufacturers, this paper explores the impacts of reselling and agency selling under various market power structures. This approach provides a more realistic perspective in light of the rapid development of e-commerce. The main research articles have been grouped, and Table 1 shows how our work fits into this literature.

**Table 1:** Summary and comparison of this paper with related studies.

Literature (year)	Green supply chain		Government subsidies	Platform economy	
	Green awareness	Marketing efforts		Power structure	Agency selling
Tian et al., 2020 [23]		√			
Xia et al., 2018 [39]	√			√	
Hong and Guo, 2019 [13]	√	√			
Cao et al., 2020 [41]	√	√		√	
Guo et al., 2020 [22]	√	√			√
Meng et al., 2021 [30]	√		√		
Chen et al., 2021 [12]				√	√
Niu et al., 2021 [42]			√	√	√
This paper	√	√	√	√	√

## 3. Model framework

### 3.1. Model descriptions

**Fig 1:**
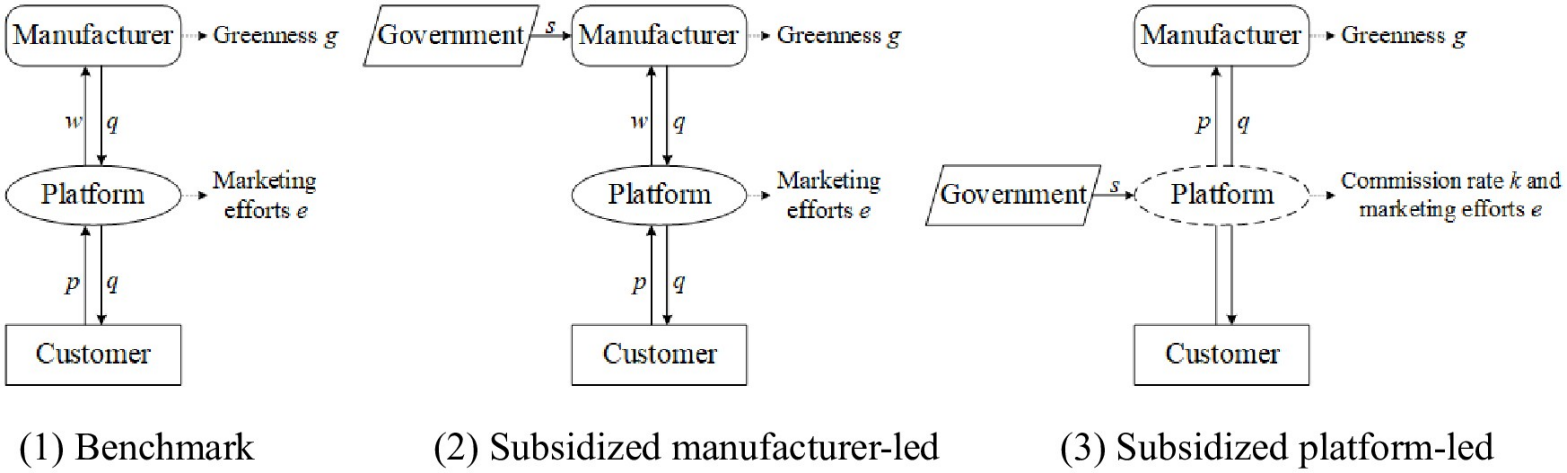
Model structure of platform supply chain carbon reduction.

In this study, a distribution channel comprising of a single manufacturer selling products through an intermediary platform is considered. The manufacturer, which dominates the supply chain, does not have its own customer base and relies on the platform to reach end customers. In the case of a platform-led supply chain, the platform enables the manufacturer to have direct access to customers, and earns revenue by charging a commission fee for each successful sale. We consider the non-subsidized manufacturer-led benchmark model (*bm*). Under the government subsidies, we consider the manufacturer-led (*sm*) and platform-led (*sp*) modes, respectively. The model structures of platform supply chain carbon reduction are shown in Fig 1.

### 3.2. Symbols and notations

To improve market competitiveness and meet the demand for environmentally friendly products, the manufacturer invests in the development of green technology to produce products with the greenness *g*, while the platform supports marketing efforts *e* to promote the product. In the presence of government subsidies, the proportion of subsidies provided to cover investment costs is denoted as *s* [14]. The e-commerce platform engage in reselling or agency selling, e-commerce platforms allow manufacturers to sell goods on their platforms at the selling price *p* and the manufacturer determines the price at which the product is sold, when platform-led the supply chain, the platform takes a proportion of the manufacturer’s online revenue with a commission rate of *k* [42]. We denote player *i*’*s* profit as $\pi_{i}^{j}$ in model *j*, where the superscript *i* = *m*, *p* represents manufacturer and platform, and the superscript *j* = *bm*, *sm*, *sp* corresponds to the benchmark model, subsidized manufacturer-led model, and subsidized platform-led model, respectively. Specifically, we summarize the symbols and notations as shown in Table 2.

**Table 2:** Symbols and notations.

Model parameters	
*α*	Consumers’ green awareness
*ν*	Consumers’ valuation
*τ*	Green technology cost coefficient
*η*	Marketing efforts cost coefficient
Decision variables	
$s_{i}^{j}$	Government subsidies proportion of investment
*k*	Commission rate
$w_{i}^{j}$	Wholesale price
$p_{i}^{j}$	Sales price
$e_{i}^{j}$	Marketing efforts
$g_{i}^{j}$	Greenness level

### 3.3. Assumption

To address the aforementioned problems, our models are based on individual consumer purchase choices. It is assumed that all consumers are heterogeneous in their value of green products, and the functional valuation *v* of green products among consumers is assumed to follow a uniform distribution in the interval [0,1] [43]. In this context, myopic consumers will purchase green products only if their utilities are non-negative. We model the consumer’s net utility as a function of the product’s price *p*, greenness *g*, the consumer’s green consciousness *α*, and marketing efforts *e*, the linear demand function, as shown in Eq (1) below, was proposed based on previous research conducted by [9, 44].


u=g(v−p+α+e)+(1−g)(v−p+e)=v−p+αg+e (1)

We assume that each consumer buys at most one unit of the product in each period. Consumers will purchase one product if their net utility is positive (i.e. *u* ≥ 0). Characterize the demand function, market demand can be derived from a consumer utility model, and the market potential is normalized to 1, the demand formula *D*(*p*, *g*, *e*) [9, 44] is given by Eq (2).


D(p,g,e)=P{v≥p−αg−e}=1−p+αg+e (2)

The investment cost of green technology has an impact on product greenness and market demand. It is assumed that the manufacturer’s investment cost for green can be expressed as a quadratic function of the greenness variable *τg*^2^/2, where *τ* is the green investment coefficient reflects the level of greenness technology [40]. In addition, consumers’ consumption consciousness directly determines market demand. To promote green products and enhance consumers’ green awareness, the platform needs to increase its investment in green marketing and allocate a significant portion of its budget to advertising. It is assumed that the cost of such marketing efforts can be expressed as a quadratic function of the variable *ηe*^2^/2, where *η* is the marketing efforts investment coefficient reflects the efficiency of marketing advertising [4]. Without loss of generality, we normalize the production cost, the selling cost, the salvage value of the product, and other fixed costs to zero [45].

The manufacturer sells products to the platform at wholesale prices and bears the investment cost of the green degree. The platform purchases products at wholesale prices and sells them to end consumers at retail prices, while bearing the investment costs of the marketing efforts. The social welfare in this model includes consumer surplus, as well as the profits of both the manufacturer and the platform, net the government subsidy expenditure [7, 46]. When the government subsidizes the manufacturer (or platform), the subsidy received is reflected in the profits of the manufacturer (or platform). Social welfare, which encompasses consumer surplus and the profits of both the manufacturer and the platform, requires the deduction of government subsidy expenditure. As such, the government subsidies within the social welfare function offset each other. Ultimately, the government subsidies are not directly represented in the final social welfare function. Therefore, the social welfare function *SW* can be expressed as follows:

$$SW=\frac{1}{2}{\left(1-p+\alpha g+e\right)}^{2}+p(1-p+\alpha g+e)-\frac{1}{2}\tau g^{2}-\frac{1}{2}\eta e^{2}$$ (3)

## 4. Model formulations and equilibrium analysis

### 4.1. Benchmark: Unsubsidized manufacturer-led

**Fig 2:**
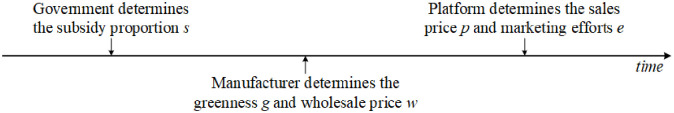
Decision sequence under the benchmark model (*bm*).

When subsidies are not provided by the government, the manufacturer-led supply chain operates in a way where the manufacturer decides the greenness and wholesale price of the product, while the platform determines the retail price and marketing efforts. The decision sequence under the benchmark model (*bm*) is shown in Fig 2. The manufacturer first determines the greenness of the product *g* and the wholesale price of the product *w*. Then the platform determines its sales price *p* and marketing efforts *e* based on the greenness and wholesale price determined by the manufacturer. Finally, the consumer decides whether to purchase the green product based on the sales price and marketing efforts.

**Lemma 1:** *Under the government’s non-subsidized and manufacturer-dominated benchmark model* (*bm*), *the equilibrium strategies for maximizing manufacturer profits are* (*w*^*bm*^)* = (*τ* − 2*ητ*)/(*α*^2^*η* + 2*τ* − 4*ητ*) *and* (*g*^*bm*^)* = −*αη*/(*α*^2^*η* + 2*τ* − 4*ητ*), *and the equilibrium strategies for maximizing platform profits are* (*p*^*bm*^)* = (*τ* − 3*ητ*)/(*α*^2^*η* + 2*τ* − 4*ητ*) *and* (*e*^*bm*^)* = −*τ*/(*α*^2^*η* + (2 − 4*η*)*τ*), *respectively*.

According to the inverse order solving rule, the platform’s retail price and marketing efforts decision are first solved. In this scenario, the platform’s profit function can be expressed as:

$$\underset{p^{bm},e^{bm}}{\text{max}}\pi_{p}^{bm}(p^{bm},e^{bm})=(p^{bm}-w^{bm})(1-p^{bm}+\alpha g^{bm}+e^{bm})-\frac{1}{2}\eta {\left(e^{bm}\right)}^{2}$$ (4)

According to Eq (4), the platform profit $\pi_{p}^{bm}(p^{bm},e^{bm})$ is a joint concave function for (*p*^*bm*^, *e*^*bm*^). The optimal retail price and marketing efforts can be expressed as follows:

$$p^{bm}=\frac{-w^{bm}+\eta \left(1+\alpha g^{bm}+w^{bm}\right)}{-1+2\eta },e^{bm}=\frac{1+\alpha g^{bm}-w^{bm}}{-1+2\eta }$$ (5)

As the leader in the supply chain, the manufacturer determines the optimal wholesale price *w* and greenness *g* based on profit maximization, given the observed platform retail price *p* and marketing efforts *e*. The profit function of the manufacturer can be expressed as follows:

$$\underset{w^{bm},g^{bm}}{\text{max}}\pi_{m}^{bm}(w^{bm},g^{bm})=w^{bm}(1-p^{bm}+\alpha g^{bm}+e^{bm})-\frac{1}{2}\tau {\left(g^{bm}\right)}^{2}$$ (6)

The platform’s optimal retail price *p*^*bm*^ and marketing efforts *e*^*bm*^ are brought into the manufacturer’s profit function, respectively. The risk-neutral manufacturer decides the optimal wholesale price *w* and greenness *g* according to profit maximization. The manufacturer’s profit $\pi_{m}^{bm}(w^{bm},g^{bm})$ is a joint concave function concerning the wholesale price *w* and the greenness *g*. Therefore, a unique set of solutions (*w*^*bm*^, *g*^*bm*^) exists that maximizes the manufacturer’s profit. The manufacturer’s optimal wholesale price *w* and greenness *g* are shown below:

$$w^{bm}=\frac{\tau -2\eta \tau }{\alpha^{2}\eta +2\tau -4\eta \tau },g^{bm}=-\frac{\alpha \eta }{\alpha^{2}\eta +2\tau -4\eta \tau }$$ (7)

Lemma 1 summarizes the equilibrium outcomes under the benchmark model (*bm*), where the optimal wholesale price *w* and greenness *g* of the manufacturer are used to update the optimal retail price *p* and marketing efforts *e* of the platform.

### 4.2. Subsidized

#### 4.2.1 Subsidized manufacturer-led

**Fig 3:**
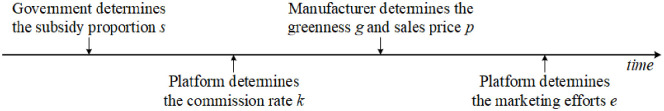
Decision sequence under the subsidized manufacturer-led model (*sm*).

In the subsidized manufacturer-led supply chain (sm) model, the decision sequence is as follows: the government determines the subsidy ratio *s* based on social welfare maximization, which is the ratio of government subsidy to the manufacturer’s green technology investment cost. Next, the manufacturer determines the optimal wholesale price *w* and greenness *g*. Later, the platform decides on the optimal retail price *p* and marketing efforts *e*, and finally, the consumer decides whether to buy the green product. Fig 3 presents the decision sequence under the subsidized manufacturer-led model (*sm*).

**Lemma 2:** *Under the subsidized and manufacturer-dominated model* (*sm*), *The equilibrium subsidy strategy that maximizes the social welfare of the government is* (*s*^*sm*^)* = (1 − 3*η*)/(3 − 7*η*), *the equilibrium wholesale price and greenness strategy that maximizes the profit of the manufacturer are* (*w*^*sm*^)* = (2(1 − 2*η*)^2^*τ*/(*α*^2^(3 − 7*η*)*η* + 4(1 − 2*η*)^2^*τ*) *and* (*g*^*sm*^)* = *α*(3 − 7*η*)*η*/(*α*^2^*η*(−3 + 7*η*) − 4(1 − 2*η*)^2^*τ*), *and the equilibrium retail price and marketing efforts strategy that maximizes platform’s profit are* (*p*^*sm*^)* = 2(1 + *η*(−5 + 6*η*))*τ*/(*α*^2^(3 − 7*η*)*η* + 4(1 − 2*η*)^2^*τ*) *and* (*e*^*sm*^)* = 2(−1 + 2*η*)*τ*/(*α*^2^(3 − 7*η*)*η* + 4(1 − 2*η*)^2^*τ*), *respectively*.

In the government-subsidized and manufacturer-dominated supply chain model (*sm*), the platform first determines the optimal retail price *p* and marketing efforts *e* using the inverse order solution rule. The profit of the risk-neutral platform can be expressed as follows:

$$\underset{p^{sm},e^{sm}}{\text{max}}\pi_{p}^{sm}(p^{sm},e^{sm})=(p^{sm}-w^{sm})(1-p^{sm}+\alpha g^{sm}+e^{sm})-\frac{1}{2}\eta {\left(e^{sm}\right)}^{2}$$ (8)

As the platform determines the optimal retail price *p* and marketing efforts *e* simultaneously, there exists a unique set of solutions (*p*^*sm*^, *e*^*sm*^) of the joint concave function for retailer price *p* and marketing efforts *e* that maximizes the platform profit $\pi_{p}^{sm}(p^{sm},e^{sm})$. The optimum *p* and *e* can be obtained as shown below:

$$p^{sm}=\frac{-w^{sm}+\eta (1+\alpha g^{sm}+w^{sm})}{-1+2\eta },e^{sm}=\frac{1+\alpha g^{sm}-w^{sm}}{-1+2\eta }$$ (9)

Based on the platform’s decision on the retail price *p* and marketing efforts *e*, the risk-neutral manufacturer determines the optimal wholesale price *w* and greenness *g*, with the goal of maximizing profit. The manufacturer’s profit can be expressed as:

$$\underset{w^{sm},g^{sm}}{\text{max}}\pi_{m}^{sm}(w^{sm},g^{sm})=w^{sm}(1-p^{sm}+\alpha g^{sm}+e^{sm})-\frac{1}{2}(1-s)\tau {\left(g^{sm}\right)}^{2}$$ (10)

Taking the platform-optimal retail price *p* and marketing efforts *e* into the manufacturer’s profit function $\pi_{m}^{sm}(w^{sm},g^{sm})$, the risk-neutral manufacturer makes decisions about the wholesale price *w* and the greenness *g* simultaneously. The manufacturer’s profit function $\pi_{m}^{sm}(w^{sm},g^{sm})$ is a joint concave function concerning the wholesale price *w* and the greenness *g*. Therefore, there exists a unique set of solutions (*w*^*sm*^, *g*^*sm*^) that maximize its profit, which can be expressed explicitly as:

$$w^{sm}=\frac{(-1+2\eta )(-1+s^{sm})\tau }{\alpha^{2}\eta +2(-1+2\eta )(-1+s^{sm})\tau },g^{sm}=-\frac{\alpha \eta }{\alpha^{2}\eta +2(-1+2\eta )(-1+s^{sm})\tau }$$ (11)

After considering the optimal decisions of both the manufacturer and the platform, the government determines the optimal subsidy rate to be given to the manufacturer for investing in green technology, with the objective of maximizing social welfare. The social welfare function is expressed as follows:

$$SW(s^{sm})=\frac{1}{2}{\left(1-p^{sm}+\alpha g^{sm}+e^{sm}\right)}^{2}+p^{sm}(1-p^{sm}+\alpha g^{sm}+e^{sm})-\frac{1}{2}\tau {\left(g^{sm}\right)}^{2}-\frac{1}{2}\eta {\left(e^{sm}\right)}^{2}$$ (12)

Taking the optimal (*w*^*sm*^, *g*^*sm*^, *p*^*sm*^, *e*^*sm*^) into the social welfare function, the social welfare function is a concave function concerning the subsidy ratio of the manufacturer’s green technology investment. The optimal government subsidy ratio can be obtained as:

$$s^{sm}=\frac{1-3\eta }{3-7\eta }$$ (13)

The optimal strategy portfolio (*w*^*sm*^, *g*^*sm*^, *p*^*sm*^, *e*^*sm*^) is updated according to the optimal government subsidy rate for the manufacturer’s green technology investment. The equilibrium strategies under the subsidized manufacturer-led model (*sm*) can be summered as Lemma 2.

#### 4.2.2 Subsidized platform-led

**Fig 4:**
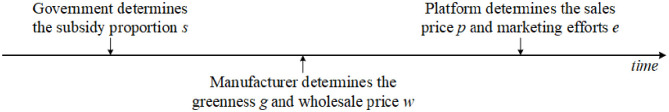
Decision sequence under the subsidized platform-led model (*sp*).

In the subsidized platform-led model (*sp*), the platform takes a proportion of the manufacturer’s online revenue with a commission rate when the platform with more substantial market power no longer retails the product. The decision-making process of the model is as follows: First, the government determines the subsidy rate *s* for the dominant platform’s marketing efforts investment, with the aim of maximizing social welfare. Next, the platform determines the commission rate *k*. Then, the risk-neutral manufacturer determines the optimal selling price *p* and greenness *g*, with the goal of maximizing profit, based on the determined commission rate and subsidy rate. Finally, the platform decides on the optimal marketing efforts *e*. The decision sequence under the subsidized platform-led model (*sp*) is shown in Fig 4.

**Lemma 3:** *Under the government subsidy and platform-led model* (*sp*)), *the equilibrium subsidy strategy that maximizes government social welfare is* (*s*^*sp*^)* = (*α*^2^(−5 + 3*η*) + 4*τ*)/(*η*(*α*^2^ + 4*τ*)), the equilibrium commission rate and marketing efforts that maximize the platform’s profit are k* = (*α*^2^ (5 − 2*η*) + 4(−1 + *η*)*τ*)/(2*α*^2^(−1 + *η*) + 8*τ*) *and* (*e*^*sp*^)* = −*τ*(*α*^2^ + 4*τ*)/((*α*^2^ − 2*τ*)(*α*^2^(−7 + 4*η*) + 4*τ*)), *and the optimal retail price and greenness that maximize the manufacturer’s profit are p*^*sp*^ = −2*τ*(*α*^2^(−1 + *η*) + 4*τ*)/((*α*^2^ − 2*τ*)(*α*^2^ (−7 + 4*η*) + 4*τ*)) *and g*^*sp*^ = (*α*^3^(7 − 4*η*) + 4*α*(− 3 + *η*)*τ*)/((*α*^2^ − 2*τ*)(*α*^2^(−7 + 4*η*) + 4*τ*)), *respectively*.

According to the inverse order-solving rule, we first solve the optimal marketing efforts of the platform *g*. The profit function of the platform can be expressed as follows.


$$\underset{e^{sp}}{\text{max}}\pi_{p}^{sp}(e^{sp})=kp^{sp}(1-p^{sp}+\alpha g^{sp}+e^{sp})-\frac{1}{2}(1-s^{sp})\eta {\left(e^{sp}\right)}^{2}$$ (14)

The platform profit is a concave function concerning the marketing efforts *e*. Let its first-order derivative be zero, so the optimal marketing efforts of the platform can be obtained as follows.


$$e^{sp}=kp^{sp}/\left(\eta -\eta s^{sp}\right)$$ (15)

Based on the optimal marketing efforts determined by the platform, the manufacturer determines the optimal selling price *p* and greenness *g*, with the goal of maximizing profit. The manufacturer’s profit function can be expressed as follows:

$$\underset{p^{sp},g^{sp}}{\text{max}}\pi_{m}^{sp}(p^{sp},g^{sp})=(1-k)p^{sp}(1-p^{sp}+\alpha g^{sp}+e^{sp})-\frac{1}{2}\tau {\left(g^{sp}\right)}^{2}$$ (16)

The manufacturer decides the optimal sales price and greenness simultaneously. The manufacturer profit function $\pi_{m}^{sp}(p^{sp},g^{sp})$ is a joint concave function for the sales price and the greenness. There is a unique set of solutions (*p*^*sp*^, *g*^*sp*^) that maximizes the manufacturer’s profit. The manufacturer’s optimal sales price and greenness are as follows.


$$p^{sp}=\frac{\eta (-1+s^{sp})\tau }{\alpha^{2}\eta (-1+k)(-1+s^{sp})+2(k+\eta (-1+s^{sp}))\tau },g^{sp}=-\frac{\alpha \eta (-1+k)(-1+s^{sp})}{\alpha^{2}\eta (-1+k)(-1+s^{sp})+2(k+\eta (-1+s^{sp}))\tau }$$ (17)

Based on the optimal selling price and greenness determined by the manufacturer, the platform determines the optimal commission rate *k*, with the goal of maximizing profit. The platform’s profit function can be expressed as follows:

$$\underset{k}{\text{max}}\pi_{p}^{sp}(k)=kp^{sp}(1-p^{sp}+\alpha g^{sp}+e^{sp})-\frac{1}{2}(1-s)\eta {\left(e^{sp}\right)}^{2}$$ (18)

The optimal marketing efforts *e*^*sp*^ of the platform and the optimal selling price *p*^*sp*^ and greenness *g*^*sp*^ of the manufacturer are brought into the platform profit function, and the platform profit function is a concave function concerning the commission rate *k*. Therefore, there exists an optimal commission rate that maximizes the platform profit, then:

$$k=-\frac{\eta (-1+s^{sp})(\alpha^{2}-2\tau )}{\alpha^{2}(3+\eta (-1+s^{sp}))-4\tau }$$ (19)

Based on the optimal decisions made by both the platform and the manufacturer, the government determines the optimal subsidy rate *s* for the platform’s marketing efforts investment, with the goal of maximizing social welfare. The social welfare function can be expressed as follows:

$$\underset{s^{sp}}{\text{max}}SW(s^{sp})=\frac{1}{2}{\left(1-p^{sp}+\alpha g^{sp}+e^{sp}\right)}^{2}+p^{sp}(1-p^{sp}+\alpha g^{sp}+e^{sp})-\frac{1}{2}\tau {\left(g^{sp}\right)}^{2}-\frac{1}{2}\eta {\left(e^{sp}\right)}^{2}$$ (20)

The optimal decisions (*k*, *e*^*sp*^, *p*^*sp*^, *g*^*sp*^) of the platform and manufacturer, respectively, are brought into the social welfare function. The social welfare function is a concave function concerning the subsidy ratio *s*. The optimal government subsidy rate can be expressed as:

$$s^{sp}=\frac{\alpha^{2}(-5+3\eta )+4\tau }{\eta (\alpha^{2}+4\tau )}$$ (21)

The optimal government subsidy rate *s*^*sp*^ is incorporated into the optimal decisions of both the platform and the manufacturer. As a result, the equilibrium decision for the subsidized and platform-dominated model (sp) is obtained, which is summarized in Lemma 3.

## 5. Analysis and numerical comparison

This section compares and analyzes the equilibrium results and profits under the three models, taking into account the impact of consumers’ green awareness, product greenness, and marketing efforts cost coefficient on marketing efforts level, product greenness, manufacturer profit, platform profit, and social welfare. The conclusions drawn from this analysis provide management with valuable insights.

The results of the model framework analysis are validated by implementing a numerical study through MATLAB to determine the impact of critical factors on the strategy results and to demonstrate the model choice by comparing the decisions and profits under the non-subsidized manufacturer-led benchmark model (*bm*), the subsidized manufacturer-led model (*sm*), and the subsidized platform-led model (*sp*). We have developed a green supply chain model comprising a single manufacturer and an e-commerce platform, examining the influence of environmental consciousness and marketing efforts on sustainability strategies. It’s worth noting that Suning.com, a significant platform retailer in China, has actively enhanced the green status of products through collaboration with upstream manufacturers such as Haier, and has driven green consumption by implementing strategic marketing efforts. Following the existing literature [47–49], we have set the consumers’ green awareness *α* = 0.9. Furthermore, we set the greening cost coefficient *τ* = 2, and the marketing effect cost coefficient *η* = 2 [50, 51]. These values are both close to real-world scenarios and satisfy the assumptions necessary for our model, including the existence of equilibrium outcomes, and positive demand and profit conditions.

### 5.1 Impact of consumers’ green awareness on equilibrium decisions

**Proposition 1:** *Under all three models* (*j = bm*, *sm*, *sp*), *as consumers’ green awareness increases*, *the manufacturer’s greenness increases* ∂*g*^*j*^/∂*α* > 0 *and always maximizes under the subsidized manufacturer-led model* (*sm*), *the platform’s marketing efforts also keep increasing* ∂*e*^*j*^/∂*α* > 0, *and the optimal marketing efforts always maximize under the subsidized platform-led model* (*sp*).

As consumers’ green awareness increases, products with higher greenness can provide more utility to consumers. As a result, more consumers are willing to pay for green products, leading to an increase in market demand for such products, which ultimately results in higher profits for the manufacturer. With higher profits and market demand, manufacturers have an incentive to produce even greener products, which leads to an increase in the manufacturer’s optimal greenness. In addition, under the subsidized manufacturer-led model *g*^*sm*^ > *g*^*sp*^ > *g*^*bm*^, the manufacturer has more market power and receives direct government subsidies for green technology investment. Hence, the manufacturer has the highest greenness in this model.

As consumers’ green awareness strengthens, the level of marketing efforts exerted by platforms also increases. This heightened marketing efforts enhance the utility for consumers, thereby boosting the demand for products. Ultimately, this leads to an increase in the platform’s profits. In addition, the platform has the highest level of green marketing efforts under the subsidized platform-led model *e*^*sp*^ > *e*^*sm*^ > *e*^*bm*^, the reason is that the platform has greater market power, uses the agency selling mode, and receives direct government subsidies. The proof is presented in S1 Appendix.

**Fig 5:**
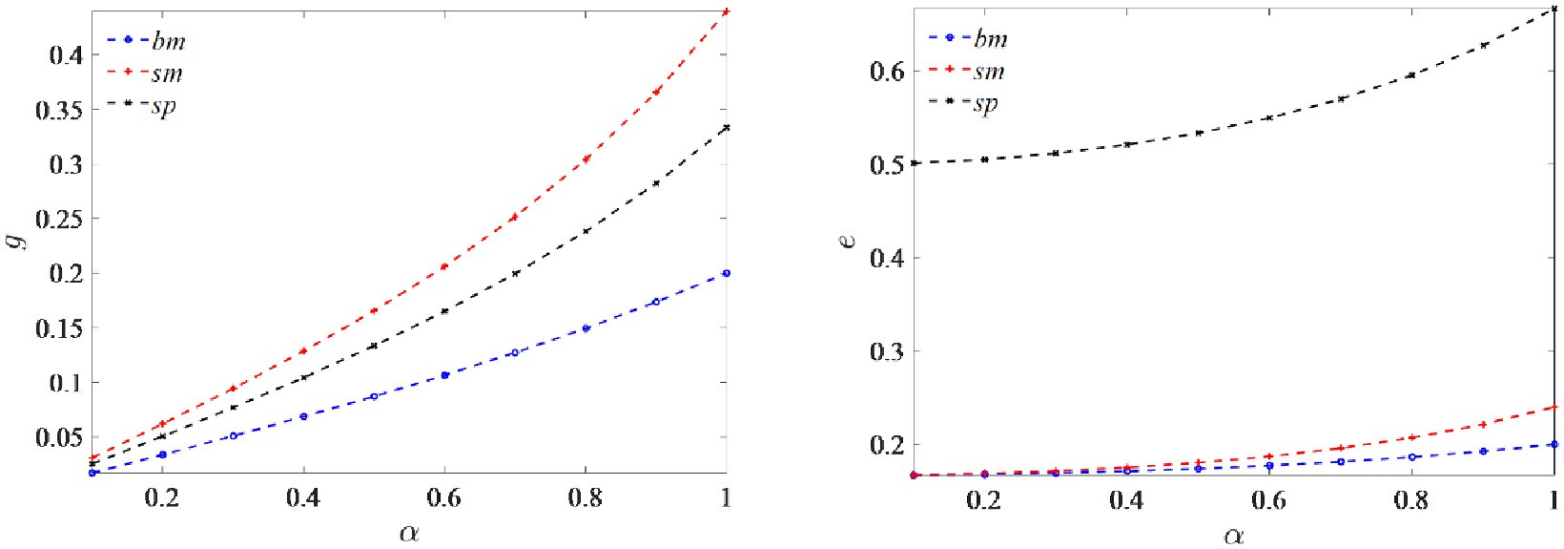
Impact of consumers’ green awareness on greenness and marketing efforts.

Fig 5 demonstrates the impact of consumers’ green awareness on greenness and marketing efforts. The possible explanations are as follows. As consumers’ green awareness increase, the manufacturer decides to increase the greenness of its products and the greenness is always maximized under the subsidized manufacturer-led model (*sm*). In contrast, the platform decides to increase its green marketing efforts and the level is always maximized under the subsidized platform-led model (*sp*). Fig 5 of the simulation validates the results of the Proposition analysis above and provides management insight. The possible reasons are as follows. As consumers’ green awareness continues to rise, particularly with the support of government subsidies, manufacturers are incentivized to continuously improve the greenness of their products to attract more consumers. The optimal strategy for manufacturers is to increase the greenness of their products and expand their customer base. Under the government subsidy, the sales platform also raise marketing efforts of green products to enhance sales, thus leading to more profits. Direct subsidies from the government can be an effective incentive for supply chain participants to increase their emissions reduction behaviour more effectively.

### 5.2 Impact of reduction investment cost coefficient on profit

**Proposition 2:** *As the manufacturer’s green technology cost coefficient τ increases*, *the manufacturer’s profit decreases for all three models and* $\pi_{m}^{sp}>\pi_{m}^{sm}>\pi_{m}^{bm}$ *always holds*. *Similarly*, *as the platform’s green marketing efforts cost coefficient η increases*, *the platform’s profit falls under the benchmark model* (*bm*) *and the subsidized manufacturer-led model* (*sm*), *while it rises under the subsidized platform-led model* (*sp*) *and always the largest* $\pi_{p}^{sp}>\pi_{p}^{sm}>\pi_{p}^{bm}$ *when η exceeds the threshold*.

As the manufacturer’s green technology cost coefficient increases, the optimal degree of greenness implemented by the manufacturer decreases to limit the investment costs. The highest degree of greenness is achieved under the subsidized manufacturer-led model (*sm*), and any reduction in greenness results in a decrease in the sales price. However, a price reduction does not necessarily prevent a contraction in demand, and therefore, the manufacturer’s profits ultimately decrease. Under the subsidized platform-led model (*sp*), the e-commerce platform engage in agency selling. The selling price and the demand for the product are the largest, contributing more to the manufacturer’s profit; therefore, the manufacturer’s profit is always maximum.

With the platform’s marketing efforts cost coefficient increasing, the platform’s optimal green marketing efforts level decreases continuously, leading to the product’s demand and the sales price eventually reducing. Consequently, the platform’s profit decreases under both the non-subsidized manufacturer-led benchmark model (*bm*) and the subsidized manufacturer-led model (*sm*), and the platform’s profit is consistently lower without government subsidies. Under the subsidized platform-led model (*sp*), although the green marketing efforts level, the sales price and the market demand all decrease, the platform’s profit increases rather than decreases. The main reason for this lies in the fact that the sales platform operates under the agency selling model where it acts as the leader and controls the performance of the supply chain by determining the commission rate. the platform increases the commission rate *k* and determines the revenue-sharing ratio. Please refer to the S1 Appendix for detailed proof.

**Fig 6:**
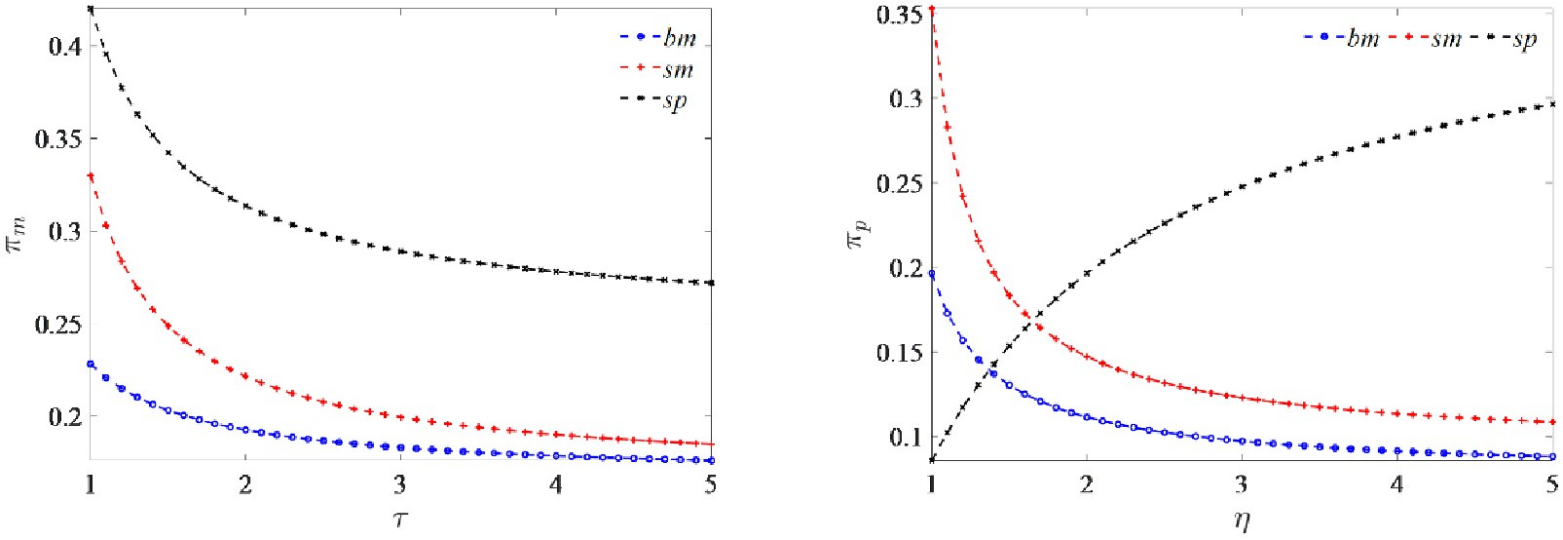
Impact of cost coefficient on profit.

Fig 6 depicts the impact of cost coefficient on profit under the three models. As the green technology cost coefficient increases, it suggests that the development of green technology has become more complicated, leading to a decrease in the capital input-output ratio, resulting in a decrease in the optimal greenness of the product. The lower greenness reduces the selling price and demand in the market, reducing the manufacturer’s profit. Furthermore, since the commission rate is almost constant under the subsidized platform-led model (*sp*), the sales price and demand for the product are the largest among all three models, leading to the most considerable profit for the manufacturer, and the rational manufacturer would choose this model.

With the increase in the marketing efforts cost coefficient, which indicates intensifying market competition, the efficiency of green marketing efforts relative to investment decreases. As a result, the optimal level of green marketing efforts exerted by the platform is likely to diminish. Furthermore, as the green marketing efforts of the product decrease, both the sales price and the demand for the product decrease correspondingly, resulting in a reduction of the platform’s profit under both the benchmark model (*bm*) and the subsidized manufacturer-led model (*sm*). However, contrary to our intuition, the platform’s profit increases under the subsidized platform-led model (*sp*) and consistently maximizes when *η* larger than the threshold compared to the other two models mainly because the platform dominates the supply chain and has adopted the agency selling mode, allowing it to increase its commission rate and boost its profit.

### 5.3 Comparison of profits in the three models

The analysis above examined the impact of individual factors on equilibrium decisions and profits. Next, we analyze the effects of multiple factors on equilibrium profit, compare the equilibrium profit under the three models, and provide optimal model selection.

**Proposition 3:** *The results of comparing profits under the three models* (*bm*, *sm*, *sp*) *are mainly determined by the green marketing efforts cost coefficient η*. *When the η is small*, *the manufacturer chooses the sp model*, *and the platform selects the sm model*. *In contrast*, *when the η is large*, *the manufacturer selects the sm model*, *and the platform determines the sp model*. *When the η is moderate*, *both the manufacturer and the platform choose the sm model*.

The green technology cost coefficient *τ* has a limited impact on the comparison of profits under the three models. The choice of model under the three models is mainly determined by the scale of the platform’s green marketing efforts cost coefficient *η*. When the platform’s green marketing efforts cost coefficient *η* is small, the manufacturer chooses the subsidized platform-led model (*sp*), the supply chain adopts the agency selling strategy in this situation, and the equilibrium level of marketing efforts, sales price and market demand are maximized, so the manufacturer’s profit reaches the maximum. The platform, on the contrary, chooses the subsidized manufacturer-led model (*sm*), mainly because the equilibrium commission rate is lower in the subsidized platform-led model (*sp*). Hence, the platform chooses to relinquish the dominance of the supply chain. Furthermore, the equilibrium outcomes under the subsidized manufacturer-led model (*sm*) are favorable and ultimately result in the highest profit for the platform. Similar explanations can be provided for other situations. The S1 Appendix provides detailed proof.

**Fig 7:**
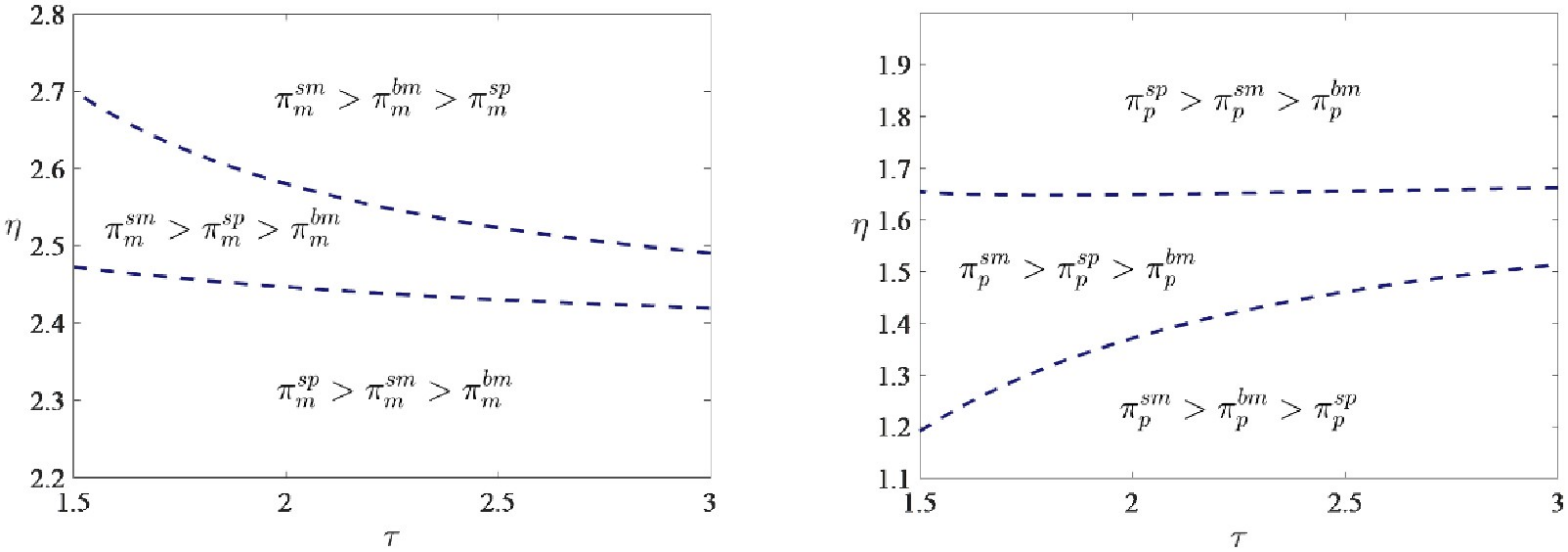
Profit comparison and model selection under three models.

Fig 7 illustrates the management insights for the profit comparison and model selection under three models. The small green marketing efforts investment cost coefficient suggests that the platform’s green marketing efforts have a more efficient input-output relationship. The smaller *η* leads to more green marketing efforts, higher sales price and market demand, consistently maximum under the subsidized platform-led model (*sp*), i.e., $\pi_{m}^{sp}>\pi_{m}^{sm}>\pi_{m}^{bm}$. Therefore, the manufacturer obtains the maximum profit under the subsidized platform-led model (*sp*). Contrary to intuition, the equilibrium commission rate *k* under the subsidized platform-led model (*sp*) is low, even though the platform’s green marketing efforts are easier and more effective to conduct, the equilibrium commission rate *k* under the subsidized platform-led model (*sp*) is low, such that $\pi_{p}^{sm}>\pi_{p}^{bm}>\pi_{p}^{sp}$, so the platform chooses the subsidized manufacturer-led model (*sm*) instead. However, the opposite conclusion is observed when the platform’s green marketing efforts cost coefficient *η* is large. Whereas, when the platform green marketing efforts cost coefficient *η* is moderate, both parties ultimately choose the subsidized manufacturer-led model (*sm*) to achieve the best situation. Since *p*^*sm*^ > *p*^*sp*^ > *p*^*bm*^ and the commission rate *k* under the subsidized platform-led model (*sp*) is neither small enough for the manufacturer to relinquish its dominance nor large enough to ensure the maximum profit of the platform.

### 5.4 Comparison of social welfare under three models

**Proposition 4:** *For any cost coefficient regarding greenness and marketing efforts*, *social welfare minimizes under the benchmark model* (*bm*) *and maximizes under the subsidized and platform-led model* (*sp*).

With the increase of the green technology cost coefficient or the marketing efforts cost coefficient, the cost of producing and marketing green products increases, which leads to a decrease in social welfare. This suggests that as the green input-output ratio decreases, the benefits of using green investment become less apparent. Moreover, the increased costs associated with being green can lead to a decline in social welfare. Therefore, it is important to find a balance between the level of greenness and the associated costs to achieve the optimal social welfare. Under the three models, the non-subsidized manufacturer-led benchmark model (*bm*) receives no government subsidies. Hence, the consumer surplus and firms’ profits are low, leading to the most inadequate social welfare. Under the subsidized platform-led model (*sp*), greenness, level of marketing efforts, sales price, and market demand are maximized, and the social welfare reaches maximum ultimately. Specific proofs can be found in the S1 Appendix.

**Fig 8:**
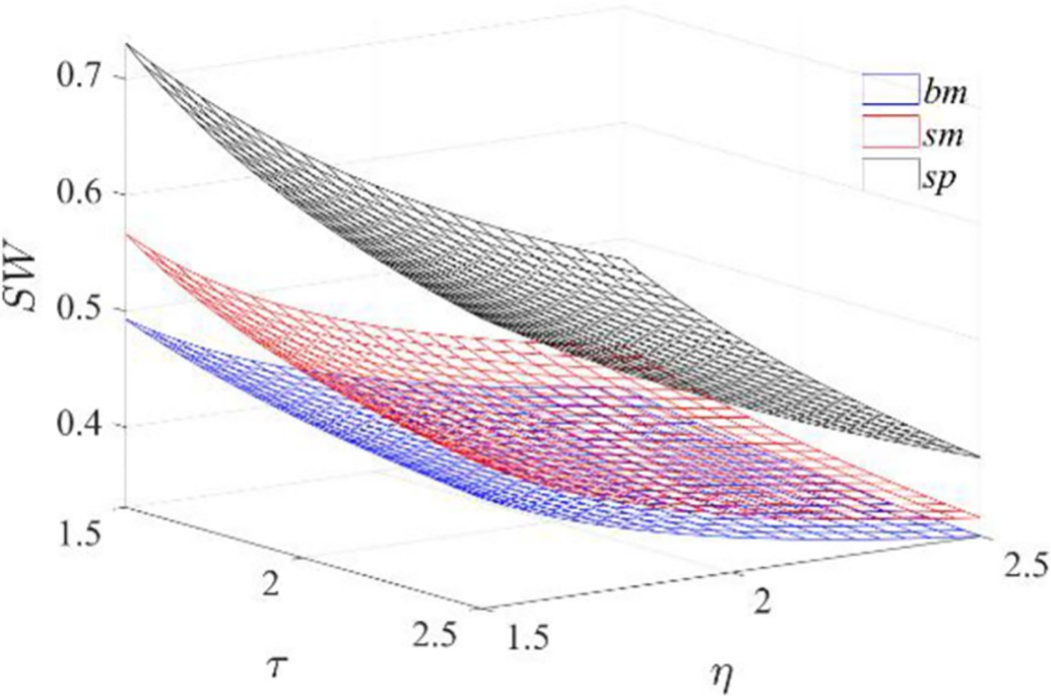
Comparison of social benefits under the three models.

The insights provided by the comparison of social benefits under the three models as depicted in Fig 8 confirm the proposition and offer guidance to the government regarding policies. It suggests that the government should promote green technology and marketing efforts to reduce investment costs, enhance the efficiency of green input-output, and increase social welfare through technological innovation and marketing strategies. Moreover, the government should encourage green subsidies, as they lead to higher social welfare. The subsidized platform-led model (*sp*) is the most favorable, followed by the agency selling mode, and the government should encourage the platform to dominate the supply chain and adopt the agency selling mode. Although the commission rate regulates the profits of the participating parties, social welfare is the highest as it leads to more significant greenness, marketing efforts level, sales price, and market demand.

## 6. Concluding remarks

The topic of green supply chain has gained significant attention due to the rising focus on environmental issues and consumers’ preference for eco-friendly products. This study examines a green supply chain comprising one manufacturer and one platform, and investigates three different models: the unsubsidized manufacturer-led model (*bm*), the subsidized manufacturer-led model (*sm*), and the subsidized platform-led model (*sp*). What makes this study unique is that, unlike existing literature on government subsidy mechanisms for a single player, the government’s proportional subsidy of investment costs for the dominant firms in the supply chain with different market power structures is considered. Additionally, the study proposes that marketing efforts for green products are a crucial factor, and the use of agency selling mode when the dominant platform is consistent with industry practices. The study aims to provide insights into the government’s subsidy strategy, the manufacturer’s greenness decision, and the platform’s level of green marketing efforts through a comparative analysis of the models. This approach provides a novel perspective on the impact of government subsidies on green supply chain and highlights the importance of marketing efforts in promoting green products. In the broader context, the greenness of products and the sustainability of supply chains are intertwined with the goal of carbon emission reduction. As industries evolve, the focus on green products will be paramount in achieving significant reductions in global carbon emissions.

The analytical results are as follows. With the rise of consumers’ green awareness, the degree of greenness and marketing efforts level can bring greater utility to consumers. Consequently, more consumers opt for green products, generating larger profits for manufacturer and platform. This inspires the manufacturer to increase its optimal greenness and platform to enhance its optimal marketing efforts level. Notably, government subsidies provide a significant stimulus. An increase in the green technology cost coefficient indicates a decrease in the efficiency of the manufacturer’s investment in green technologies, this leads to a decline in the manufacturer’s profits across all three modes. Intriguingly, the manufacturer’s profits are always highest in the *sp* mode, the primary reason is that the platform exerts the most significant marketing efforts in the *sp* mode (as Proposition 1), resulting in more sales. As the marketing efforts cost coefficient increases, it indicates a drop in the efficiency of the platform’s marketing efforts, leading to a reduction in the equilibrium level of marketing efforts. As a result, the platform’s profits decrease in the *bm* and *sm* modes. In contrast, in the *sp* mode, the platform, due to its dominant position in the supply chain, opts to increase its commission rate, which results in a profit increase rather than a decrease. Comparative analysis of the three strategic modes reveals that the choice of mode primarily depends on the platform’s marketing efforts cost coefficient. When this coefficient exceeds a threshold, the platform chooses the *sp* mode (as Proposition 2). However, due to the relatively low marketing efficiency in this scenario, the manufacturer prefers the *sm* mode. From a social welfare perspective, for the government, the *sp* mode involves agency selling, serves as an effective mechanism for the government to redistribute subsidies, thereby yielding the maximum social welfare benefits.

This research offers managerial insights for supply chain managers and policymakers regarding government subsidies. As consumers become increasingly environmentally conscious, the eco-friendliness of products and dedicated marketing efforts drive demand for eco-friendly goods. Therefore, prudent manufacturers should enhance the green attributes of their products, while platforms should intensify their marketing efforts to align with changing market trends. Both manufacturers and platforms should strive to improve investment efficiency to minimize costs. When the marketing efforts cost coefficient is low, suggesting high marketing efficiency, there is a corresponding high equilibrium marketing level and increased product demand. In such scenarios, manufacturers should opt for the *sp* mode to maximize profits. Contrary to intuition, the *sp* mode doesn’t necessarily yield the highest profit for platforms at this juncture due to the lower equilibrium commission rate (as Lemma 1–3). Thus, platforms might be better off choosing the *sm* mode. For policymakers crafting government subsidy strategies, subsidizing leading platforms in the green supply chain can maximize societal welfare. Under these circumstances, platforms employ the agency selling mode, effectively reallocating the subsidies.

This paper has some limitations that provide potential directions for future research. Firstly, the study only considers a green supply chain with one manufacturer and one platform, and it can be extended to multiple manufacturers or multiple platform scenarios in the future. Secondly, for the sake of simplicity in the model and clarity of conclusions, the study assumes deterministic consumer demand, and future research can consider stochastic demand or different green preferences of consumers. Lastly, government interventions, besides subsidies, are also crucial research directions that can be explored.

## Supporting information

S1 Appendix (DOCX)
