# Supplementary material for: Sustainable strategies for green supply chain within the platform economy consider subsidies and marketing efforts
Source: PLoS One. 2023 Nov 28;18(11):e0292349. doi: 10.1371/journal.pone.0292349 (PMC10684003; doi:10.1371/journal.pone.0292349)
Supplement: S1 Appendix — (DOCX) [file pone.0292349.s001.docx]

# Appendix

## Proof of Lemma 1

According to the inverse order solving rule, we first solve the platform's retail price and marketing efforts decision. In this scenario, the profit function of the platform can be expressed as:

| $\max_{p^{bm},e^{bm}} \pi_{p}^{bm}(p^{bm},e^{bm})=(p^{bm}-w^{bm})(1-p^{bm}+\alpha g^{bm}+e^{bm})-\frac{1}{2}\eta(e^{bm})^{2}$ | (A1) |
| --- | --- |

According to Equation (A1), the first and second-order derivatives of the profit function of the platform concerning the retail price *p* are $\partial\pi_{p}^{bm}/\partial p^{bm}=1+e^{bm}+\alpha g^{bm}-2p^{bm}+w^{bm}$ and $\partial^{2}\pi_{p}^{bm}/\partial(p^{bm})^{2}=-2<0$, respectively. The first and second-order derivatives of the profit function of the platform concerning the marketing efforts *e* are $\partial\pi_{p}^{bm}/\partial e^{bm}=-\eta e^{bm}+p^{bm}-w^{bm}$ and $\partial^{2}\pi_{p}^{bm}/\partial(e^{bm})^{2}=-\eta<0$, respectively, and the mixed partial derivative of the platform concerning the *p* and *e* is obtained easily $\partial^{2}\pi_{p}^{bm}/\partial p^{bm}\partial e^{bm}=\partial^{2}\pi_{p}^{bm}/\partial e^{bm}\partial p^{bm}=1$.

Thus, the second-order Hesse matrix of the platform profit function concerning the retail price *p* and the marketing efforts *e* is shown below.

$$H=\left[\begin{matrix} \frac{\partial^{2}\pi_{p}^{bm}}{\partial(p^{bm})^{2}} & \frac{\partial^{2}\pi_{p}^{bm}}{\partial p^{bm}\partial e^{bm}} \\ \frac{\partial^{2}\pi_{p}^{bm}}{\partial e^{bm}\partial p^{bm}} & \frac{\partial^{2}\pi_{p}^{bm}}{\partial(e^{bm})^{2}} \end{matrix}\right]=\left[\begin{matrix} -2 & 1 \\ 1 & -\eta\end{matrix}\right]$$

Since the first-order principle subformula corresponding to the second-order Hesse matrix is negative $-2<0$ and the second-order principle subformula $2\eta-1>0$ when $\eta>1/2$, so the second-order Hesse matrix is negative definite, there exists a unique set of retail price and marketing efforts that maximize platform profits. Thus, the platform profit $\pi_{p}^{bm}(p^{bm},e^{bm})$ is a joint concave function concerning $(p^{bm},e^{bm})$. Let the first-order derivatives of the platform profit concerning *p* and *e* be zero, i.e., $\partial\pi_{p}^{bm}/\partial p^{bm}\text{=0}$ and $\partial\pi_{p}^{bm}/\partial e^{bm}\text{=0}$, respectively. The joint Equation gives the optimal retail price and marketing efforts.

|  | $\begin{matrix} p^{bm}=\frac{-w^{bm}+\eta\left(1+\alpha g^{bm}+w^{bm}\right)}{-1+2\eta} \\ e^{bm}=\frac{1+\alpha g^{bm}-w^{bm}}{-1+2\eta} \end{matrix}$ | (A2) |
| --- | --- | --- |

The manufacturer, as the leader, decides the optimal wholesale price *w* and greenness *g* based on profit maximization after the observed platform retail price *p* and marketing efforts *e*. The manufacturer’s profit function can be expressed as follows:

|  | $\max_{w^{bm},g^{bm}} \pi_{m}^{bm}(w^{bm},g^{bm})=w^{bm}(1-p^{bm}+\alpha g^{bm}+e^{bm})-\frac{1}{2}\tau(g^{bm})^{2}$ | (A3) |
| --- | --- | --- |

The platform's optimal retail price $p^{bm}$ and marketing efforts $e^{bm}$ are brought into the manufacturer’s profit function, respectively. The risk-neutral manufacturer decides the optimal wholesale price *w* and greenness *g* according to profit maximization. The first and second-order derivatives of the manufacturer’s profit concerning the wholesale price *w* are $\partial\pi_{m}^{bm}/\partial w^{bm}=\eta(1+\alpha g^{bm}-2w^{bm})/(-1+2\eta)$ and $\partial^{2}\pi_{m}^{bm}/\partial(w^{bm})^{2}=2\eta/(1-2\eta)<0$, respectively, the first and second-order derivatives of the manufacturer’s profit concerning the greenness *g* are $\partial\pi_{m}^{bm}/\partial g^{bm}=\alpha\eta/(-1+2\eta)$ and $\partial^{2}\pi_{m}^{bm}/\partial(g^{bm})^{2}=-\tau$, respectively, and the mixed partial derivative of the manufacturer’s profit concerning *w* and *g* is $\partial^{2}\pi_{m}^{bm}/\partial w^{bm}\partial g^{bm}=\partial^{2}\pi_{m}^{bm}/\partial g^{bm}\partial w^{bm}=\alpha\eta/(-1+2\eta)$.

Thus, the second-order Hesse matrix of the manufacturer’s profit function concerning the wholesale price *w* and the greenness *g* is shown below.

$$H=\left[\begin{matrix} \frac{\partial^{2}\pi_{m}^{bm}}{\partial(w^{bm})^{2}} & \frac{\partial^{2}\pi_{m}^{bm}}{\partial w^{bm}\partial g^{bm}} \\ \frac{\partial^{2}\pi_{m}^{bm}}{\partial g^{bm}\partial w^{bm}} & \frac{\partial^{2}\pi_{m}^{bm}}{\partial(g^{bm})^{2}} \end{matrix}\right]=\left[\begin{matrix} \frac{2\eta}{1-2\eta} & \frac{\alpha\eta}{-1+2\eta} \\ \frac{\alpha\eta}{-1+2\eta} & -\tau\end{matrix}\right]$$

The first-order principal subequation corresponding to the second-order Hessian matrix of the manufacturer’s profit function concerning the wholesale price *w* and the greenness *g* is negative $2\eta/(1-2\eta)<0$ when $\eta>1/2$, and its second-order principal subequation is positive $-\eta(\alpha^{2}\eta+(2-4\eta)\tau)/(1-2\eta)^{2}>0$ when $\eta>2\tau/(4\tau-\alpha^{2})$. Thus manufacturer’s profit $\pi_{m}^{bm}(w^{bm},g^{bm})$ is a joint concave function concerning the wholesale price *w* and the greenness *g*. Therefore, there exists a unique set of solutions $(w^{bm},g^{bm})$ that maximizes the manufacturer’s profit. The optimal wholesale price *w* and greenness *g* are obtained from the joint equations by making the first-order derivative of the manufacturer’s profit concerning the wholesale price *w* be zero $\partial\pi_{m}^{bm}/\partial w^{bm}\text{=0}$, and the first-order derivative concerning the greenness *g* be zero $\partial\pi_{m}^{bm}/\partial g^{bm}\text{=0}$, respectively. The manufacturer’s optimal wholesale price *w* and greenness *g* are shown below:

|  | $\begin{matrix} w^{bm}=\frac{\tau-2\eta\tau}{\alpha^{2}\eta+2\tau-4\eta\tau} \\ g^{bm}=-\frac{\alpha\eta}{\alpha^{2}\eta+2\tau-4\eta\tau} \end{matrix}$ | (A4) |
| --- | --- | --- |

Based on the manufacturer’s optimal wholesale price *w* and greenness *g*, the platform’s optimal retail price *p* and marketing efforts *e* are updated. The equilibrium outcomes under the benchmark model (*bm*) are summered as follows.

|  | $\begin{matrix} w^{bm}=\frac{\tau-2\eta\tau}{\alpha^{2}\eta+2\tau-4\eta\tau} \\ g^{bm}=-\frac{\alpha\eta}{\alpha^{2}\eta+2\tau-4\eta\tau} \\ p^{bm}=\frac{\tau-3\eta\tau}{\alpha^{2}\eta+2\tau-4\eta\tau} \\ e^{bm}=-\frac{\tau}{\alpha^{2}\eta+\left(2-4\eta\right)\tau} \end{matrix}$ | (A5) |
| --- | --- | --- |

Summarizing the above analysis, we can get Lemma 1.

## Proof of Lemma 2

When the government subsidizes and the manufacturer dominates the supply chain (*sm*), the platform first decides the optimal retail price *p* and marketing efforts *e* according to the inverse order solution rule, and the profit of the risk-neutral platform can be expressed as follows:

|  | $\max_{p^{sm},e^{sm}} \pi_{p}^{sm}(p^{sm},e^{sm})=(p^{sm}-w^{sm})(1-p^{sm}+\alpha g^{sm}+e^{sm})-\frac{1}{2}\eta(e^{sm})^{2}$ | (A6) |
| --- | --- | --- |

Since the platform simultaneously decides the optimal retail price p and marketing efforts e, the second-order Hessian matrix of the platform profit concerning *p* and *e* needs to be constructed. The first and second-order derivatives of platform profit concerning retail price *p* are $\partial\pi_{p}^{sm}/\partial p^{sm}=1+e^{sm}+\alpha g^{sm}-2p^{sm}+w^{sm}$ and $\partial^{2}\pi_{p}^{sm}/\partial(p^{sm})^{2}=-2$, respectively, and the first and second-order derivatives of platform profit concerning marketing efforts *e* are $\partial\pi_{p}^{sm}/\partial e^{sm}=-e^{sm}\eta+p^{sm}-w^{sm}$ and $\partial^{2}\pi_{p}^{sm}/\partial(e^{sm})^{2}=-\eta<0$, respectively. The mixed partial derivatives of platform profit function concerning retail price p and marketing efforts *e* can be expressed as $\partial^{2}\pi_{p}^{sm}/\partial p^{sm}\partial e^{sm}=\partial^{2}\pi_{p}^{sm}/\partial e^{sm}\partial p^{sm}=1$.

Thus, the second-order Hessian matrix of platform profit concerning retail price *p* and marketing efforts *e* can be expressed as:

$$H=\left[\begin{matrix} \frac{\partial^{2}\pi_{p}^{sm}}{\partial(p^{sm})^{2}} & \frac{\partial^{2}\pi_{p}^{sm}}{\partial p^{sm}\partial e^{sm}} \\ \frac{\partial^{2}\pi_{p}^{sm}}{\partial e^{sm}\partial p^{sm}} & \frac{\partial^{2}\pi_{p}^{sm}}{\partial(e^{sm})^{2}} \end{matrix}\right]=\left[\begin{matrix} -2 & 1 \\ 1 & -\eta\end{matrix}\right]$$

Since the second-order Hessian matrix corresponds to the first-order master equation $-2<0$ and the second-order master equation $2\eta-1>0$ when $\eta>1/2$, therefore, there exists a unique set of solutions $(p^{sm},e^{sm})$ of the joint concave function concerning retailer price *p* and marketing efforts *e* that maximizes the platform profit $\pi_{p}^{sm}(p^{sm},e^{sm})$, let the first-order derivative of the platform profit concerning the retailer price be zero $\partial\pi_{p}^{sm}/\partial p^{sm}\text{=0}$, and the first-order derivative of the platform profit concerning the marketing efforts be zero $\partial\pi_{p}^{sm}/\partial e^{sm}\text{=0}$, respectively, and solve the equations to obtain the optimal p and e as follows.

|  | $\begin{matrix} p^{sm}=\frac{-w^{sm}+\eta(1+\alpha g^{sm}+w^{sm})}{-1+2\eta} \\ e^{sm}=\frac{1+\alpha g^{sm}-w^{sm}}{-1+2\eta} \end{matrix}$ | (A7) |
| --- | --- | --- |

Based on the retail price *p* and marketing efforts *e* of the platform decision, the risk-neutral manufacturer determines the optimal wholesale price *w* and greenness *g* based on profit maximization, and the manufacturer’s profit can be expressed as:

|  | $\max_{w^{sm},g^{sm}} \pi_{m}^{sm}(w^{sm},g^{sm})=w^{sm}(1-p^{sm}+\alpha g^{sm}+e^{sm})-\frac{1}{2}(1-s)\tau(g^{sm})^{2}$ | (A8) |
| --- | --- | --- |

Taking the platform-optimal retail price *p* and marketing efforts *e* into the manufacturer’s profit function$\pi_{m}^{sm}(w^{sm},g^{sm})$, the risk-neutral manufacturer makes decisions about the wholesale price *w* and the greenness *g* simultaneously. The first-order and second-order derivatives of the manufacturer’s profit concerning the wholesale price *w* are $\partial\pi_{m}^{sm}/\partial w^{sm}=\eta(1+\alpha g^{sm}-2w_{sw})/(-1+2\eta)$ and $\partial^{2}\pi_{m}^{sm}/\partial(w^{sm})^{2}=2\eta/(1-2\eta)$, respectively, and the first-order and second-order derivatives of the manufacturer’s profit function concerning the greenness are $\partial\pi_{m}^{sm}/\partial g^{sm}=(g^{sm}(-1+s^{sm})\tau+\alpha\eta w^{sm})/(-1+2\eta)$ and $\partial^{2}\pi_{m}^{sm}/\partial(g^{sm})^{2}=(-1+s^{sm})\tau$, respectively. The second-order mixed partial derivative of the manufacturer’s profit function concerning the wholesale price *w* and the greenness *g* is $\partial^{2}\pi_{m}^{sm}/\partial w^{sm}\partial g^{sm}=\partial^{2}\pi_{m}^{sm}/\partial w^{sm}\partial g^{sm}=\alpha\eta/-1+2\eta$, so that the second-order Hessian matrix of the manufacturer’s profit function concerning the wholesale price w and the greenness is obtained as:

$$H=\left[\begin{matrix} \frac{\partial^{2}\pi_{m}^{sm}}{\partial(w^{sm})^{2}} & \frac{\partial^{2}\pi_{m}^{sm}}{\partial w^{sm}\partial g^{sm}} \\ \frac{\partial^{2}\pi_{m}^{sm}}{\partial g^{sm}\partial w^{sm}} & \frac{\partial^{2}\pi_{m}^{sm}}{\partial(g^{sm})^{2}} \end{matrix}\right]=\left[\begin{matrix} \frac{2\eta}{1-2\eta} & \frac{\alpha\eta}{-1+2\eta} \\ \frac{\alpha\eta}{-1+2\eta} & (-1+s^{sm})\tau\end{matrix}\right]$$

Since the first-order rank principal subequation corresponding to the second-order Hesse matrix is $2\eta/(1-2\eta)<0$ when $\eta>1/2$ and the second-order rank principal subequation is $-\eta(\alpha^{2}\eta+2(-1+2\eta)(-1+s^{sm})\tau)/(1-2\eta)^{2}>0$ when $\eta>2(1-s^{sm})\tau/(4(1-s^{sm})\tau-\alpha^{2})$, the manufacturer’s profit function $\pi_{m}^{sm}(w^{sm},g^{sm})$ is a joint concave function concerning the wholesale price *w* and the greenness *g*. Therefore, there exists a unique set of solutions $(w^{sm},g^{sm})$ that maximize its profit. Also, let the first-order partial derivative of the manufacturer profit function concerning the wholesale price w be zero $\partial\pi_{m}^{sm}/\partial w^{sm}\text{=0}$, and let the first-order partial derivative of the manufacturer profit function concerning the greenness g be zero $\partial\pi_{m}^{sm}/\partial g^{sm}\text{=0}$, solving the system of equations yields the manufacturer’s optimal wholesale price and greenness, which can be expressed explicitly as:

|  | $\begin{matrix} w^{sm}=\frac{(-1+2\eta)(-1+s^{sm})\tau}{\alpha^{2}\eta+2(-1+2\eta)(-1+s^{sm})\tau} \\ g^{sm}=-\frac{\alpha\eta}{\alpha^{2}\eta+2(-1+2\eta)(-1+s^{sm})\tau} \end{matrix}$ | (A9) |
| --- | --- | --- |

Based on the optimal decision of the manufacturer and the platform, the government decides the optimal subsidy rate given to the manufacturer to invest in green technology based on the objective of maximizing social welfare, and the social welfare function is expressed as:

|  | $SW(s^{sm})=\frac{1}{2}(1-p^{sm}+\alpha g^{sm}+e^{sm})^{2}+p^{sm}(1-p^{sm}+\alpha g^{sm}+e^{sm})-\frac{1}{2}\tau(g^{sm})^{2}-\frac{1}{2}\eta(e^{sm})^{2}$ | (A10) |
| --- | --- | --- |

Taking the optimal $(w^{sm},g^{sm},p^{sm},e^{sm})$ into the social welfare function, the first and second-order derivatives of the social welfare function concerning the subsidy ratio are expressed, respectively.

$$\begin{matrix} \frac{\partial SW(s^{sm})}{\partial s^{sm}}\text{=}\frac{\alpha^{2}\eta^{2}(1-3s^{sm}+\eta(-3+7s^{sm}))\tau^{2}}{(\alpha^{2}\eta+2(-1+2\eta)(-1+s^{sm})\tau)^{3}} \\ \frac{\partial^{2}SW(s^{sm})}{\partial(s^{sm})^{2}}\text{=}\frac{\alpha^{2}\eta^{2}\tau^{2}(\alpha^{2}\eta(-3+7\eta)-4(-1+2\eta)(-3s^{sm}+\eta(-1+7s^{sm}))\tau)}{(\alpha^{2}\eta+2(-1+2\eta)(-1+s^{sm})\tau)^{4}} \end{matrix}$$

The social welfare function is a concave function concerning the subsidy ratio of the manufacturer’s green technology investment, and we make the first-order derivative of the social welfare function concerning the subsidy ratio of zero $\partial SW(s^{sm})/\partial s^{sm}\text{=0}$, the optimal government subsidy ratio can be obtained as:

|  | $s^{sm}=\frac{1-3\eta}{3-7\eta}$ | (A11) |
| --- | --- | --- |

The optimal strategy portfolio $(w^{sm},g^{sm},p^{sm},e^{sm})$ is updated according to the optimal government subsidy rate for the manufacturer’s green technology investment so that the equilibrium strategies under the subsidized manufacturer-led model (*sm*) can be obtained.

|  | $\begin{matrix} s^{sm}=\frac{1-3\eta}{3-7\eta} \\ w^{sm}=\frac{2(1-2\eta)^{2}\tau}{\alpha^{2}(3-7\eta)\eta+4(1-2\eta)^{2}\tau} \\ g^{sm}=\frac{\alpha(3-7\eta)\eta}{\alpha^{2}\eta(-3+7\eta)-4(1-2\eta)^{2}\tau} \\ p^{sm}=\frac{2(1+\eta(-5+6\eta))\tau}{\alpha^{2}(3-7\eta)\eta+4(1-2\eta)^{2}\tau} \\ e^{sm}=\frac{2(-1+2\eta)\tau}{\alpha^{2}(3-7\eta)\eta+4(1-2\eta)^{2}\tau} \end{matrix}$ | (A12) |
| --- | --- | --- |

Summarizing the above analysis, we can get Lemma 2.

## Proof of Lemma 3

According to the inverse order solving rule, we first solve the optimal marketing efforts of the platform *g*. The profit function of the platform can be expressed as follows.

|  | $\max_{e^{sp}} \pi_{p}^{sp}(e^{sp})=kp^{sp}(1-p^{sp}+\alpha g^{sp}+e^{sp})-\frac{1}{2}(1-s^{sp})\eta(e^{sp})^{2}$ | (A13) |
| --- | --- | --- |

The first-order derivative of the platform profit concerning the marketing efforts is $\partial\pi_{p}^{sp}/\partial e^{sp}=kp^{sp}-\eta e^{sp}(1-s^{sp})$ and the second-order derivative is $\partial^{2}\pi_{p}^{sp}/\partial(e^{sp})^{2}=-\eta e^{sp}(1-s^{sp})<0$. Hence, the platform profit is a concave function concerning the marketing efforts *e*. Let its first-order derivative be zero, the optimal marketing efforts of the platform can be obtained as follows.

|  | $e^{sp}=kp^{sp}/(\eta-\eta s^{sp})$ | (A14) |
| --- | --- | --- |

According to the optimal marketing efforts of the platform, the manufacturer decides the optimal selling price *p* and greenness *g* based on profit maximization, and the manufacturer’s profit function can be expressed as follows:

|  | $\max_{p^{sp},g^{sp}} \pi_{m}^{sp}(p^{sp},g^{sp})=(1-k)p^{sp}(1-p^{sp}+\alpha g^{sp}+e^{sp})-\frac{1}{2}\tau(g^{sp})^{2}$ | (A15) |
| --- | --- | --- |

The manufacturer decides the optimal sales price and greenness simultaneously. The first-order and second-order derivatives of the manufacturer profit function concerning the sales price *p* are $\partial\pi_{m}^{sp}/\partial p^{sp}=(-1+k)(-1-\alpha g^{sp}+p^{sp}(2+(2k/(-\eta+\eta s^{sp}))))$ and $\partial^{2}\pi_{m}^{sp}/\partial(p^{sp})^{2}=2(1-k)(-1+k/(\eta-\eta s^{sp}))$, and the first-order and second-order derivatives of the manufacturer profit function concerning the greenness *g* are $\partial\pi_{m}^{sp}/\partial g^{sp}=\alpha(p^{sp}-kp^{sp})-g^{sp}\tau$ and $\partial^{2}\pi_{m}^{sp}/\partial(g^{sp})^{2}=-\tau<0$, respectively, and the second-order mixed partial derivatives of the manufacturer profit function concerning the sales price and greenness is $\partial^{2}\pi_{m}^{sp}/\partial p^{sp}\partial g^{sp}=\partial^{2}\pi_{m}^{sp}/\partial g^{sp}\partial p^{sp}=(1-k)\alpha$, so the second-order Hessian matrix of the manufacturer profit function concerning the sales price and greenness can be obtained as follows:

$$H=\left[\begin{matrix} \frac{\partial^{2}\pi_{m}^{sp}}{\partial(p^{sp})^{2}} & \frac{\partial^{2}\pi_{m}^{sp}}{\partial p^{sp}\partial g^{sp}} \\ \frac{\partial^{2}\pi_{m}^{sp}}{\partial g^{sp}\partial p^{sp}} & \frac{\partial^{2}\pi_{m}^{sp}}{\partial(g^{sp})^{2}} \end{matrix}\right]=\left[\begin{matrix} 2(1-k)(-1+\frac{k}{\eta-\eta s^{sp}}) & (1-k)\alpha\\ (1-k)\alpha& -\tau\end{matrix}\right]$$

The first-order principal subformula of the determinant corresponds to the second-order Hessian matrix $2(1-k)(-1+k/(\eta-\eta s^{sp}))<0$, when $\eta>k/(1-s^{sp})$, and the second-order principle subformula $2\tau(1-k/(\eta-\eta s^{sp}))-(1-k)\alpha^{2}>0$, when $\eta>2\tau k/(1-s^{sp})(2\tau-(1-k)\alpha^{2})$. Therefore, the second-order Hessian matrix is negative definite, and the manufacturer profit function $\pi_{m}^{sp}(p^{sp},g^{sp})$is a joint concave function concerning the sales price and the greenness. There exists a unique set of solutions $(p^{sp},g^{sp})$ that maximizes the manufacturer profit. Let the first-order derivatives of the manufacturer profit function concerning the sales price $\partial\pi_{m}^{sp}/\partial p^{sp}=(-1+k)(-1-\alpha g^{sp}+p^{sp}(2+2k/\eta(-1+s^{sp})))$ and the first-order derivatives of the manufacturer profit function concerning the greenness $\partial\pi_{m}^{sp}/\partial g^{sp}\text{=}\alpha(p^{sp}-kp^{sp})-\tau g^{sp}$ be zero, respectively, and solve the equations to obtain the manufacturer’s optimal sales price and greenness as follows.

|  | $\begin{matrix} p^{sp}=\frac{\eta(-1+s^{sp})\tau}{\alpha^{2}\eta(-1+k)(-1+s^{sp})+2(k+\eta(-1+s^{sp}))\tau} \\ g^{sp}=-\frac{\alpha\eta(-1+k)(-1+s^{sp})}{\alpha^{2}\eta(-1+k)(-1+s^{sp})+2(k+\eta(-1+s^{sp}))\tau} \end{matrix}$ | (A16) |
| --- | --- | --- |

According to the manufacturer’s optimal sales price and greenness, the platform determines the optimal commission rate *k*, and the profit function of the platform can be expressed at this time as:

|  | $\max_{k} \pi_{p}^{sp}(k)=kp^{sp}(1-p^{sp}+\alpha g^{sp}+e^{sp})-\frac{1}{2}(1-s)\eta(e^{sp})^{2}$ | (A17) |
| --- | --- | --- |

The optimal marketing efforts $e^{sp}$ of the platform and the optimal selling price $p^{sp}$ and greenness $g^{sp}$ of the manufacturer are brought into the platform profit function, respectively. Then the first and second-order derivatives of the platform profit concerning the commission rate *k* are obtained as:

$$\begin{matrix} \frac{\partial\pi_{p}^{sp}}{\partial k}=-\frac{\eta^{2}(-1+s^{sp})^{2}\tau^{2}(\alpha^{2}(3k+\eta(1+k)(-1+s^{sp}))+2(\eta-2k-\eta s^{sp})\tau)}{(\alpha^{2}\eta(-1+k)(-1+s^{sp})+2(k+\eta(-1+s^{sp}))\tau)^{3}} \\ \frac{\partial^{2}\pi_{p}^{sp}}{\partial k^{2}}=\frac{\left(\begin{matrix} \eta^{2}(-1+s^{sp})^{2}\tau^{2}(\alpha^{4}\eta(3+6k+2\eta(2+k)(-1+s^{sp}))(-1+s^{sp})- \\ 4\alpha^{2}(-3k+\eta(1+k+2\eta(-1+s^{sp}))(-1+s^{sp}))\tau+4(\eta-4k-\eta s^{sp})\tau^{2}) \end{matrix}\right)}{(\alpha^{2}\eta(-1+k)(-1+s^{sp})+2(k+\eta(-1+s^{sp}))\tau)^{4}} \end{matrix}$$

Thus, the platform profit function is a concave function concerning the commission rate *k*. Therefore, there exists an optimal commission rate that maximizes the platform profit, such that $\partial\pi_{p}^{sp}/\partial k=0$, then:

|  | $k=-\frac{\eta(-1+s^{sp})(\alpha^{2}-2\tau)}{\alpha^{2}(3+\eta(-1+s^{sp}))-4\tau}$ | (A18) |
| --- | --- | --- |

According to the optimal decisions of the platform and the manufacturer, the government determines the optimal subsidy rate s for the platform marketing investment based on the principle of social welfare maximization, and the social welfare function can be expressed as:

|  | $\max_{s^{sp}} SW(s^{sp})=\frac{1}{2}(1-p^{sp}+\alpha g^{sp}+e^{sp})^{2}+p^{sp}(1-p^{sp}+\alpha g^{sp}+e^{sp})-\frac{1}{2}\tau(g^{sp})^{2}-\frac{1}{2}\eta(e^{sp})^{2}$ | (A19) |
| --- | --- | --- |

The optimal decisions $(k,e^{sp},p^{sp},g^{sp})$ of the platform and manufacturer, respectively, are brought into the social welfare function. Then the social welfare function is evaluated for the first-order and second-order derivatives concerning the subsidy rate, respectively, as follows:

$$\begin{matrix} \frac{\partial SW(s^{sp})}{\partial s^{sp}}=-\frac{\alpha^{2}\eta\tau^{2}(\alpha^{2}(5+\eta(-3+s^{sp}))+4(-1+\eta s^{sp})\tau)}{(\alpha^{2}-2\tau)(\alpha^{2}(3+2\eta(-1+s^{sp}))-2\tau)^{3}} \\ \frac{\partial^{2}SW(s^{sp})}{\partial(s^{sp})^{2}}=\frac{\alpha^{2}\eta^{2}\tau^{2}(\alpha^{4}(27+4\eta(-4+s^{sp}))+2\alpha^{2}(-17+\eta(4+8s^{sp}))\tau+8\tau^{2})}{(\alpha^{2}-2\tau)(\alpha^{2}(3+2\eta(-1+s^{sp}))-2\tau)^{4}} \end{matrix}$$

Thus, the social welfare function is a concave function concerning the subsidy ratio *s*. Therefore, there exists a subsidy rate that maximizes social welfare by satisfying the condition $\partial SW(s^{sp})/\partial s^{sp}=0$, and thus the optimal government subsidy rate can be expressed as:

|  | $s^{sp}=\frac{\alpha^{2}(-5+3\eta)+4\tau}{\eta(\alpha^{2}+4\tau)}$ | (A20) |
| --- | --- | --- |

The optimal government subsidy rate $s^{sp}$ is brought into the optimal decision of the platform and the manufacturer, respectively, so that the equilibrium decision under the subsidized and platform-dominated model (*sp*) is obtained and can be expressed as:

|  | $\begin{matrix} s^{sp}=\frac{\alpha^{2}(-5+3\eta)+4\tau}{\eta(\alpha^{2}+4\tau)} \\ k=\frac{\alpha^{2}(5-2\eta)+4(-1+\eta)\tau}{2\alpha^{2}(-1+\eta)+8\tau} \\ g^{sp}=\frac{\alpha^{3}(7-4\eta)+4\alpha(-3+\eta)\tau}{(\alpha^{2}-2\tau)(\alpha^{2}(-7+4\eta)+4\tau)} \\ p^{sp}=-\frac{2\tau(\alpha^{2}(-1+\eta)+4\tau)}{(\alpha^{2}-2\tau)(\alpha^{2}(-7+4\eta)+4\tau)} \\ e^{sp}=-\frac{\tau(\alpha^{2}+4\tau)}{(\alpha^{2}-2\tau)(\alpha^{2}(-7+4\eta)+4\tau)} \end{matrix}$ | (A21) |
| --- | --- | --- |

Summarizing the above analysis, we can get Lemma 3.

## Proof of Proposition 1

According to the above mentioned $\alpha>0$, $\tau>0$, $\eta>0$, under the benchmark model (*bm*), according to the equilibrium outcome in Lemma 1 (A5), we know the equilibrium greenness and marketing efforts are $g^{bm}=-\alpha\eta/(\alpha^{2}\eta+2\tau-4\eta\tau)$, $e^{bm}\text{=}-\tau/\alpha^{2}\eta+(2-4\eta)\tau$, respectively. The first-order derivative of $g^{bm}$ and $e^{bm}$ concerning the customers’ green awareness $\alpha$ can be expressed as:

|  | $\begin{matrix} \frac{\partial g^{bm}}{\partial\alpha}=\frac{\eta(\alpha^{2}\eta-2\tau+4\eta\tau)}{(\alpha^{2}\eta+\left(2-4\eta\right)\tau)^{2}} \\ \frac{\partial e^{bm}}{\partial\alpha}=\frac{2\alpha\eta\tau}{(\alpha^{2}\eta+\left(2-4\eta\right)\tau)^{2}} \end{matrix}$ | (A22) |
| --- | --- | --- |

It’s easy to get $\partial e^{bm}/\partial\alpha\text{=}2\alpha\eta\tau/(\alpha^{2}\eta+(2-4\eta)\tau)^{2}>0$, when $\eta>2\tau/(\alpha^{2}+4\tau)$, $\partial g^{bm}/\partial\alpha=\eta(\alpha^{2}\eta-2\tau+4\eta\tau)/(\alpha^{2}\eta+\left(2-4\eta\right)\tau)^{2}>0$.Therefore, under the manufacturer-led benchmark model (*bm*), the manufacturer’s greenness and the platform’s marketing efforts rise with the improvement of consumers’ green awareness.

Under the subsidized manufacturer-led model (*sm*), in the equilibrium outcome in Lemma 2 (A12), the manufacturer’s equilibrium greenness, $g^{sm}=\alpha(3-7\eta)\eta/(\alpha^{2}\eta(-3+7\eta)-4(1-2\eta)^{2}\tau)$, the equilibrium of marketing efforts, $e^{sm}=2(-1+2\eta)\tau/(\alpha^{2}(3-7\eta)\eta+4(1-2\eta)^{2}\tau)$.

|  | $\begin{matrix} \frac{\partial g^{sm}}{\partial\alpha}=\frac{\eta(-3+7\eta)(\alpha^{2}\eta(-3+7\eta)+4(1-2\eta)^{2}\tau)}{(\alpha^{2}\eta(-3+7\eta)-4(1-2\eta)^{2}\tau)^{2}} \\ \frac{\partial e^{sm}}{\partial\alpha}=-\frac{4\alpha(3-7\eta)\eta(-1+2\eta)\tau}{(\alpha^{2}(3-7\eta)\eta+4(1-2\eta)^{2}\tau)^{2}} \end{matrix}$ | (A23) |
| --- | --- | --- |

According to the above equilibrium formula, when $\eta(-3+7\eta)(\alpha^{2}\eta(-3+7\eta)+4(1-2\eta)^{2}\tau)>0$, then $\partial g^{sm}/\partial\alpha>0$; when $4\alpha\eta\tau(3-7\eta)(-1+2\eta)<0$, then $\partial e^{sm}/\partial\alpha>0$. Under the manufacturer-led benchmark model (*sm*), the manufacturer’s greenness rises with the improvement of consumers’ green awareness. The platform’s marketing efforts grow with the improvement of consumers' green awareness.

Under the subsidized platform-led model (*sp*), with the equilibrium outcome in Lemma 3 (A21), the manufacturer’s greenness, $g^{sp}=\alpha^{3}(7-4\eta)+4\alpha(-3+\eta)\tau/(\alpha^{2}-2\tau)(\alpha^{2}(-7+4\eta)+4\tau)$, the equilibrium of marketing efforts, $e^{sp}=-\tau(\alpha^{2}+4\tau)/((\alpha^{2}-2\tau)(\alpha^{2}(-7+4\eta)+4\tau))$, and the first-order derivative of $g^{sp}$ and $e^{sp}$ concerning the customers’ green awareness can be expressed as:

|  | $\begin{matrix} \frac{\partial g^{sp}}{\partial\alpha}=\frac{\begin{matrix} \alpha^{6}(7-4\eta)^{2}-2\alpha^{4}(-9+2\eta)(-7+4\eta)\tau\\ +8\alpha^{2}\tau^{2}(6+\eta(-9+4\eta))-32\tau^{3}(-3+\eta) \end{matrix}}{(\alpha^{2}-2\tau)^{2}(\alpha^{2}(-7+4\eta)+4\tau)^{2}} \\ \frac{\partial e^{sp}}{\partial\alpha}=\frac{2\alpha\tau((-7+4\eta)(\alpha^{4}+8\alpha^{2}\tau)+16(5-2\eta)\tau^{2})}{(\alpha^{2}-2\tau)^{2}(\alpha^{2}(-7+4\eta)+4\tau)^{2}} \end{matrix}$ | (A24) |
| --- | --- | --- |

When $\alpha^{6}(7-4\eta)^{2}-2\alpha^{4}(-9+2\eta)(-7+4\eta)\tau+8\alpha^{2}\tau^{2}(6+\eta(-9+4\eta))-32\tau^{3}(-3+\eta)>0$, then$\partial g^{sp}/\partial\alpha>0$, when $2\alpha\tau((-7+4\eta)(\alpha^{4}+8\alpha^{2}\tau)+16(5-2\eta)\tau^{2})>0$, the first derivative of $e^{sp}$ is positive. Therefore, under the manufacturer-led benchmark model (*sp*), the manufacturer’s greenness and the platform’s marketing efforts rise with the improvement of consumers’ green awareness.

Summarizing the above analysis, we can get Proposition 1.

## Proof of Proposition 2

According to the equilibrium results in Lemma 1, taking the equilibrium decision $w^{bm}$, $g^{bm}$, $p^{bm}$, $e^{bm}$ into the profit function of the platform and manufacturer in the benchmark model (*bm*), we can obtain the equilibrium profits of the manufacturer and the platform $\pi_{m}^{bm}$, $\pi_{p}^{bm}$ respectively. Similarly, taking the equilibrium results $s^{sm}$, $w^{sm}$, $g^{sm}$, $p^{sm}$, $e^{sm}$ in Lemma 2 into the profit function of the manufacturer and the platform in the subsidized manufacturer-led model (*sm*), we obtain the equilibrium profits of the manufacturer and the platform $\pi_{m}^{sm}$, $\pi_{p}^{sm}$ respectively. Furthermore, taking the equilibrium results $s^{sp}$， $k$， $g^{sp}$， $p^{sp}$， $e^{sp}$ in Lemma 3 into the profit function of the manufacturer and the platform in the subsidized platform-led model (*sp*), we obtain the equilibrium profits of the manufacturer and the platform $\pi_{m}^{sp}$, $\pi_{p}^{sp}$respectively. To summarize the above analysis, the equilibrium profits of the manufacturer and the platform under the three models (*bm, sm, sp*) can be expressed as:

|  | $\begin{matrix} \pi_{p}^{bm}=\frac{\eta(-1+2\eta)^{2}\tau^{2}}{2(\alpha^{2}\eta+(2-4\eta)\tau)^{2}} \\ \pi_{m}^{bm}=-\frac{\eta\tau}{2\alpha^{2}\eta+4\tau-8\eta\tau} \\ \pi_{p}^{sm}=\frac{2\eta(-1+2\eta)^{3}\tau^{2}}{(\alpha^{2}\eta(-3+7\eta)-4(1-2\eta)^{2}\tau)^{2}} \\ \pi_{m}^{sm}=\frac{\eta(-1+2\eta)\tau}{\alpha^{2}(3-7\eta)\eta+4(1-2\eta)^{2}\tau} \\ \pi_{p}^{sp}=\frac{\tau^{2}(\alpha^{2}(5-2\eta)+4(-1+\eta)\tau)}{2(\alpha^{2}-2\tau)^{2}(\alpha^{2}(-7+4\eta)+4\tau)} \\ \pi_{m}^{sp}=\frac{\tau(\alpha^{2}(7-4\eta)+4(-3+\eta)\tau)}{2(\alpha^{2}-2\tau)(\alpha^{2}\left(-7+4\eta\right)+4\tau)} \end{matrix}$ | (A25) |
| --- | --- | --- |

The first-order derivative of the manufacturer’s equilibrium profit with respect to the green technology cost coefficient $\tau$ under the three models (*bm, sm, sp*) can be expressed as:

$$\begin{matrix} \frac{\partial\pi_{m}^{bm}}{\partial\tau}=-\frac{\alpha^{2}\eta^{2}}{2(\alpha^{2}\eta+(2-4\eta)\tau)^{2}} \\ \frac{\partial\pi_{m}^{sm}}{\partial\tau}=-\frac{\alpha^{2}\eta^{2}(-1+2\eta)(-3+7\eta)}{(\alpha^{2}\eta(-3+7\eta)-4(1-2\eta)^{2}\tau)^{2}} \\ \frac{\partial\pi_{m}^{sp}}{\partial\tau}=-\frac{\left(\begin{matrix} \alpha^{6}(7-4\eta)^{2}-8\alpha^{4}(-3+\eta)(-7+4\eta)\tau\\ +8\alpha^{2}(20+\eta(-17+4\eta))\tau^{2} \end{matrix}\right)}{2(\alpha^{2}-2\tau)^{2}(\alpha^{2}(-7+4\eta)+4\tau)^{2}} \end{matrix}$$

According to the first derivative under the three models, It is easy to prove that $\partial\pi_{m}^{bm}/\partial\tau<0$, $\partial\pi_{m}^{sm}/\partial\tau<0$, $\partial\pi_{m}^{sp}/\partial\tau<0$, the main reason lies in the consumers’ green awareness $\alpha>0$, greenness cost coefficient $\tau>0$, and the marketing efforts cost coefficient $\eta>0$. Therefore, under the three models (*bm, sm, sp*), the manufacturer’s profit reduces with the rise of the green technology cost coefficient $\tau$.

The first-order derivatives of the platform’s equilibrium profit with respect to the marketing efforts cost coefficient under the three models (*bm, sm, sp*) can be expressed as follows:

$$\begin{matrix} \frac{\partial\pi_{p}^{bm}}{\partial\eta}=\frac{\tau^{2}(\alpha^{2}\eta-2\tau+4\eta\tau)}{2(\alpha^{2}\eta+\left(2-4\eta\right)\tau)^{3}} \\ \frac{\partial\pi_{p}^{sm}}{\partial\eta}=\frac{2\tau^{2}(3\alpha^{2}(1-2\eta)^{2}\eta(-1+3\eta)+4(1-2\eta)^{4}\tau)}{(\alpha^{2}\eta(-3+7\eta)-4(1-2\eta)^{2}\tau)^{3}} \\ \frac{\partial\pi_{p}^{sp}}{\partial\eta}=-\frac{(3\alpha^{2}-2\tau)\tau^{2}(\alpha^{2}+4\tau)}{(\alpha^{2}-2\tau)^{2}(\alpha^{2}(-7+4\eta)+4\tau)^{2}} \end{matrix}$$

It is obtained that $\partial\pi_{p}^{bm}/\partial\eta<0$, when the condition$(2-4\eta)\tau/\alpha^{2}<\eta<-(2-4\eta)\tau/\alpha^{2}$ holds. In addition, $\partial\pi_{p}^{sm}/\partial\eta<0$ when the condition $\tau>-3\alpha^{2}(1-2\eta)^{2}\eta(-1+3\eta)/4(1-2\eta)^{4}$ holds. Thus, the platform’s equilibrium profit decreases concerning the marketing efforts cost coefficient in the benchmark model (*bm*) and the subsidized manufacturer-led model (*sm*). However, the platform’s equilibrium profit is an increasing function of the cost coefficient of the marketing efforts $\partial\pi_{p}^{sp}/\partial\eta>0$, when the condition $\tau>3\alpha^{2}/2$ is satisfied.

Summarizing the above analysis, we can get Proposition 2.

## Proof of Proposition 3

Since the impact of green technology cost coefficient $\tau$ on model selection is limited, we focus on analyzing the impact of marketing efforts cost coefficient $\eta$ on decision making. According to Proposition 2, we have investigated the relationship between the platform's equilibrium profit function and the marketing efforts cost coefficient. Then we explore the relationship between the manufacturer's equilibrium profit function and the marketing efforts cost coefficient under the three models (*bm, sm, sp*), which can be expressed as follows.

$$\begin{matrix} \frac{\partial\pi_{m}^{bm}}{\partial\eta}=-\frac{\tau^{2}}{(\alpha^{2}\eta+(2-4\eta)\tau)^{2}} \\ \frac{\partial\pi_{m}^{sm}}{\partial\eta}=-\frac{\tau(\alpha^{2}\eta^{2}+4(1-2\eta)^{2}\tau)}{(\alpha^{2}\eta(-3+7\eta)-4(1-2\eta)^{2}\tau)^{2}} \\ \frac{\partial\pi_{m}^{sp}}{\partial\eta}=\frac{2\tau^{2}(\alpha^{2}+4\tau)}{(\alpha^{2}-2\tau)(\alpha^{2}(-7+4\eta)+4\tau)^{2}} \end{matrix}$$

According to the first-order derivative of the manufacturer's equilibrium profit with respect to the marketing efforts cost coefficient $\eta$. Therefore, it can be obtained that $\partial\pi_{m}^{bm}/\partial\eta<0$, $\partial\pi_{m}^{sm}/\partial\eta<0$, and in addition $\partial\pi_{m}^{sp}/\partial\eta<0$, when $\alpha^{2}/2<\tau$. Thus, as the marketing efforts cost coefficient grows, the equilibrium profit of the manufacturer decreases under the three models (*bm*, *sm*, sp) when the condition is satisfied.

Summarizing the above analysis, we can get Proposition 3.

## Proof of Proposition 4

Taking the equilibrium results $(k,e^{j},p^{j},g^{j})$ into the social welfare function, and the social welfare function under the three modes (*j*=*bm, sm, sp*) can be expressed as:

|  | $\begin{matrix} SW^{bm}=\frac{\eta\tau(-\alpha^{2}\eta+(-3+7\eta)\tau)}{2(\alpha^{2}\eta+(2-4\eta)\tau)^{2}} \\ SW^{sm}=\frac{(3-7\eta)\eta\tau}{2\alpha^{2}\eta(-3+7\eta)-8(1-2\eta)^{2}\tau} \\ SW^{sp}=\frac{\tau(\alpha^{4}(7-4\eta)+\alpha^{2}(-23+11\eta)\tau-4(-5+\eta)\tau^{2})}{2(\alpha^{2}-2\tau)^{2}(\alpha^{2}\left(-7+4\eta\right)+4\tau)} \end{matrix}$ | (A26) |
| --- | --- | --- |

The equilibrium social welfare $SW^{j}$ for each of the three models (*j*= *bm, sm, sp*) regarding the green technology and marketing efforts cost coefficients to obtain the first-order derivatives. It can be expressed as follows:

$$\begin{matrix} \frac{\partial SW^{bm}}{\partial\eta}=\frac{\tau^{2}(-\alpha^{2}\eta+2(-3+8\eta)\tau)}{2(\alpha^{2}\eta+(2-4\eta)\tau)^{3}} \\ \frac{\partial SW^{bm}}{\partial\tau}=-\frac{\alpha^{2}\eta^{2}(\alpha^{2}\eta+2(2-5\eta)\tau)}{2(\alpha^{2}\eta+(2-4\eta)\tau)^{3}} \\ \frac{\partial SW^{sm}}{\partial\eta}=-\frac{2(-1+2\eta)(-3+8\eta)\tau^{2}}{(\alpha^{2}\eta(-3+7\eta)-4(1-2\eta)^{2}\tau)^{2}} \\ \frac{\partial SW^{sm}}{\partial\tau}=-\frac{\alpha^{2}(3-7\eta)^{2}\eta^{2}}{2(\alpha^{2}(3-7\eta)\eta+4(1-2\eta)^{2}\tau)^{2}} \\ \frac{\partial SW^{sp}}{\partial\eta}=-\frac{\tau^{2}(\alpha^{2}+4\tau)^{2}}{2(\alpha^{2}-2\tau)^{2}(\alpha^{2}(-7+4\eta)+4\tau)^{2}} \\ \frac{\partial SW^{sp}}{\partial\tau}=\frac{\left(\begin{matrix} -\alpha^{8}(7-4\eta)^{2}+2\alpha^{6}(-7+4\eta)(-16+7\eta)\tau\\ -16\alpha^{4}(25+\eta(-19+3\eta))\tau^{2}+32\alpha^{2}(8+(-5+\eta)\eta)\tau^{3} \end{matrix}\right)}{2(\alpha^{2}-2\tau)^{3}(\alpha^{2}(-7+4\eta)+4\tau)^{2}} \end{matrix}$$

Under the benchmark model (*bm*), $\partial SW^{bm}/\partial\eta<0$, when $-\alpha^{2}\eta/(2-4\eta)<\tau<\alpha^{2}\eta/2(-3+8\eta)$ holds, and $\partial SW^{bm}/\partial\tau<0$, when the condition $(4\eta-2)\tau/\alpha^{2}>\eta$ is satisfied. Similarly, we can obtain $\partial SW^{sm}/\partial\eta<0$, $\partial SW^{sm}/\partial\tau<0$, under the subsidized manufacturer-led model (*sm*), and $\partial SW^{sp}/\partial\eta<0$, $\partial SW^{sp}/\partial\tau<0$, under the subsidized platform-led model (*sp*). Furthermore, we compare the equilibrium social welfare under the three models with $SW^{bm}<SW^{sm}<SW^{sp}$ for any green technology and marketing efforts cost coefficients.

Summarizing the above analysis, we can get Proposition 4.
